# Forced treadmill running and ladder climbing physical activity attenuates cognitive decline and protein aggregates in aged 3xTg-AD mice

**DOI:** 10.1186/s13041-026-01332-z

**Published:** 2026-07-23

**Authors:** Mingming Yang, Xiaosen Ouyang, Sishir Gautam, Malique Singleton, Viyaleta Davydzenka, Jianhua Zhang, Yun Li

**Affiliations:** 1 Department of Zoology and Physiology, University of Wyoming, 1000E University Avenue, Laramie, WY 82071 USA; 2 Department of Pathology, University of Alabama at Birmingham, Birmingham, AL 35294 USA

**Keywords:** Alzheimer’s disease, Age, Amyloid-beta, Behavior, Neuroinflammation, Protein aggregates, Inhibitory neurons

## Abstract

Alzheimer’s disease (AD) is characterized by a progressive cognitive decline and pathological cascade involving amyloid-β (Aβ) plaque, hyperphosphorylated tau (pTau), neuroinflammation, and neurodegeneration. Although anti-amyloid antibody therapies have recently been approved, their clinical benefits remain limited and often accompanied with significant adverse effects. Physical activity has emerged as a promising non-pharmacological intervention for AD, with both aerobic and resistance physical activity showing beneficial effects in preclinical studies. However, the effects of different types of physical activity remain insufficiently understood. In this study, we investigated the effects of a forced treadmill running and ladder climbing physical activity procedure on cognitive behaviors, gliosis, and protein aggregate pathology in aged 3xTg-AD mice. Thirteen to fourteen months old 3xTg-AD mice were subjected to a six-week forced treadmill running and ladder climbing physical activity protocol. Cognitive functions were evaluated using the novel object recognition, Y-maze, and social behavior tests. Hippocampal and cortical tissues were analyzed by immunostaining for Aβ, pTau, Glial Fibrillary Acidic Protein (GFAP), Ionized Calcium-Binding Adapter Molecule 1 (IBA1), and inhibitory neuron markers. Our study demonstrated that the six-week forced treadmill running and ladder climbing physical activity procedure reduced age-associated cognitive declines in spatial working memory, object recognition memory, and sociability in aged 3xTg-AD mice. Such physical activity procedure also reduced Thioflavin S (Thio S) labeled misfolded protein aggregates and reactive gliosis in the cortex and/or hippocampus. Notably, forced treadmill running and ladder climbing physical activity procedure decreased Thio S positive neuropathology within somatostatin-positive interneurons of the hippocampus and cortex, indicating a cell-specific neuroprotective effect. Our results demonstrated that forced treadmill running and ladder climbing physical activity procedure produced significant behavioral and neuropathological benefits in aged 3xTg-AD mice, supporting physical activity as a promising non-pharmacological intervention for modulating gliosis and protein aggregate pathology in aging and AD.

**Supplementary Information:**

The online version contains supplementary material available at https://doi.org/10.1186/s13041-026-01332-z.

## Introduction

Alzheimer’s disease (AD) is a progressive neurodegenerative disorder characterized by cognitive decline and pathological accumulations of amyloid-β (Aβ) and hyperphosphorylated tau (pTau) [1, 2]. Neuroinflammation occurs and further impairs protein clearance, disrupts synaptic homeostasis, and drives neuronal dysfunction [3, 4]. In addition to these global pathological changes, AD also affects network stability *via* selectively targeting vulnerable neuronal populations such as parvalbumin (PV)-positive inhibitory interneurons [5–7] and somatostatin (SST)-positive inhibitory interneurons [7–13]. Loss or dysfunction in these interneurons has been linked to disrupted excitation-inhibition balance and impaired memory-related circuit [14–16].

While monoclonal antibody therapies targeting Aβ have received Food and Drug Administration (FDA) approval, their limited clinical benefits and severe side effects [17] make alternative therapeutic strategies including non-pharmacological interventions remain urgently needed. Recent studies indicate that lifestyle changes provide benefits on cognition, consistent with preclinical studies demonstrating the benefits of physical activity in AD models [18]. Various studies in animal models suggest that different types of physical activity interventions boost hippocampal neurogenesis and reduce the neuropathological hallmarks of AD, thereby protecting against cognitive decline [19, 20]. Both voluntary and involuntary aerobic exercise trainings (e.g., voluntary wheel running and forced treadmill running) have been shown to exert protective effects on AD mouse models, such as improving Aβ clearance, increasing hippocampal volumes, attenuating spatial memory impairment, and improving recognition memory [20–26]. Aging is often accompanied by sarcopenia, a decline in muscle mass and strength, which would limit the capacity for aerobic activities [27]. Resistance exercise trainings, involving muscle contractions against external resistance, potentially addresses this issue by improving muscular strength and endurance [28]. Emerging evidence suggests that resistance exercise trainings not only combat sarcopenia but also confer cognitive benefits [29]. Studies have demonstrated that resistance exercise trainings can enhance executive function, increase brain-derived neurotrophic factor (BDNF) levels, and reduce neuroinflammation [30]. Despite the distinct benefits of aerobic and resistance exercise trainings [31], the effects of combination of different types of physical activity on age-associated cognitive decline and pathology remain insufficiently understood.

Here we investigated the effects of a forced treadmill running and ladder climbing physical activity procedure on cognition and pathology on aged 3xTg-AD mice, a mouse model harboring three human mutations (APPSwedish, PSEN1M146V, and MAPTP301L) and displaying both AD pathological hallmarks on Aβ plaques and neurofibrillary tau tangles [32]. We demonstrated that the six-week forced treadmill running and ladder climbing physical activity procedure protected against age-associated cognitive declines in spatial working memory and object recognition memory, and preserved sociability in aged 3xTg-AD mice. We also performed a suite of immunostaining analyses to complement behavioral findings. We demonstrated that the six-week forced treadmill running and ladder climbing physical activity procedure reduced cortical misfolded protein aggregates identified by Thioflavin S (Thio S) staining and reduced reactive gliosis in the hippocampus and cortex. Importantly, we observed decreased Thio S positive neuropathology specifically within SST-positive interneurons in the hippocampus and cortex, indicating a cell-specific neuroprotective mechanism with forced treadmill running and ladder climbing physical activity. Through the integration of behavioral and molecular analyses, our study supports the beneficial effects of forced treadmill running and ladder climbing physical activity procedure on cognitive performance and neuropathology in the aged 3xTg-AD mouse model.

## Results

### A six-week forced treadmill running and ladder climbing physical activity procedure on aged 3xTg-AD mice

We aimed to investigate effects of a six-week forced treadmill running and ladder climbing physical activity procedure on the cognition and pathology of aged 3xTg-AD mice, a mouse model harboring three human mutations (APPSwedish, PSEN1M146V, and MAPTP301L) and displaying age-related cognitive deficits and neuropathological changes on Aβ plaques and neurofibrillary tangles that mirror those observed in human AD [32]. 3xTg-AD mice displayed the onset of learning and memory deficits at 4 months of age [33], amyloid plaques and gliosis at 6–7 months of age [32, 34], and Tau tangles at 12 months of age [32].

**Fig. 1:**
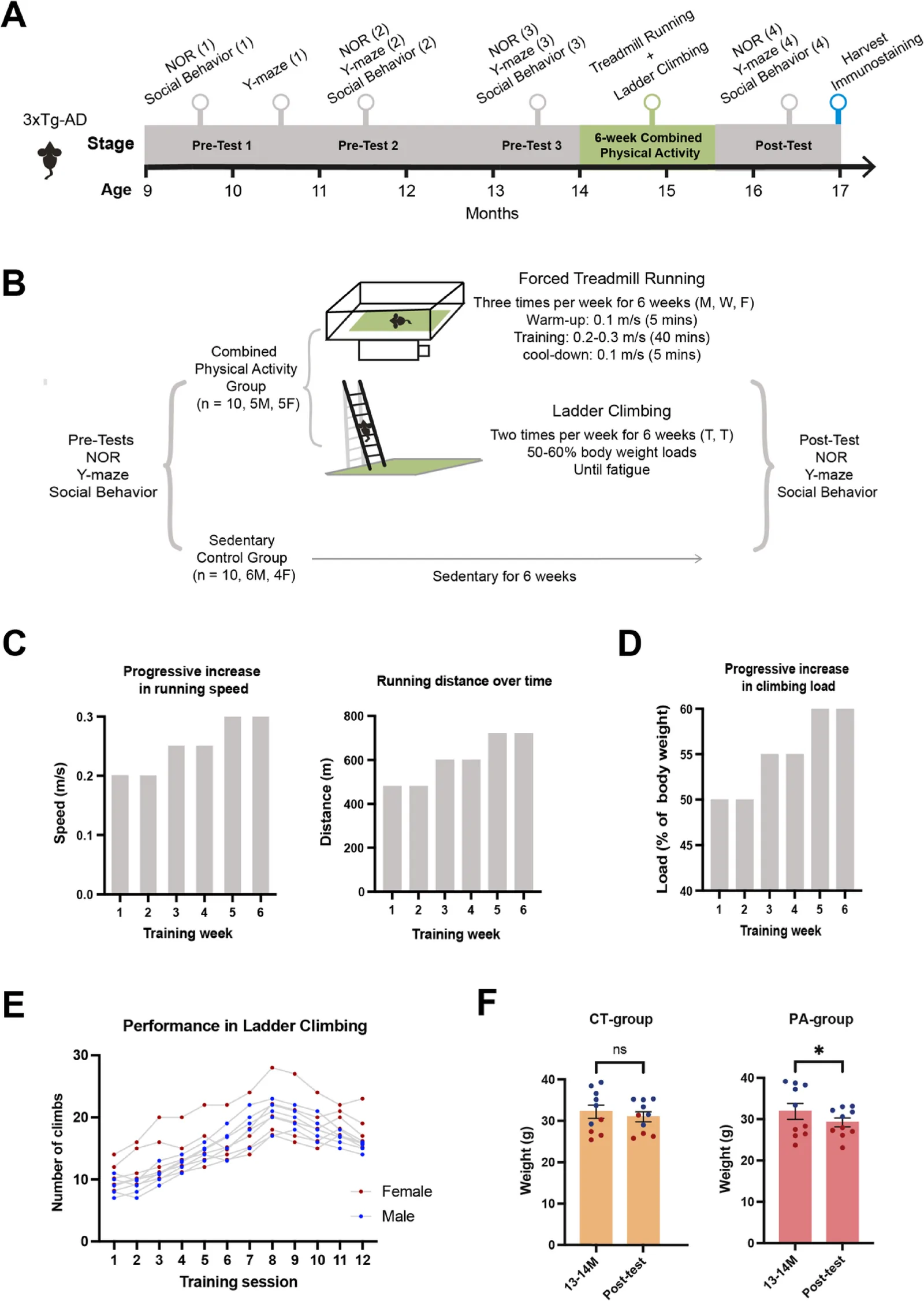
Experimental design and progression of the forced treadmill running and ladder climbing physical activity intervention. **A** Schematic overview of the experimental timeline. Behavioral assessments, including novel object recognition (NOR), Y-maze, and social behavior tests, were performed at three pre-test time points corresponding to 9–11, 11–12, and 13–14 months of age. Mice then underwent a 6-week forced treadmill running and ladder climbing physical activity intervention. Post-test behavioral assessments, including NOR, Y-maze, and social behavior tests, were conducted at 16–17 months of age, followed by tissue collection for immunostaining. **B** Schematic illustration of the experimental groups. Mice were assigned to either the forced treadmill running and ladder climbing physical activity group (PA-group; n = 10, 5 males and 5 females) or the sedentary control group (CT-group; n = 10, 6 males and 4 females). The PA-group performed forced treadmill running three times per week for 6 weeks, with each session including a 5-min warm-up at 0.1 m/s, 40 min of training at 0.2–0.3 m/s, and a 5-min cool-down at 0.1 m/s. Ladder-climbing physical activity was performed twice per week for 6 weeks, with progressive loads corresponding to 50–60% of body weight until fatigue. The CT-group remained sedentary for the same 6-week period. Behavior tests were conducted before and after the intervention. **C** Bar graphs indicating the designed forced treadmill running protocol. Left panel showing the progressive increase in forced treadmill running speed across the 6-week training period. Running speed was gradually increased from 0.2 m/s to 0.3 m/s over the course of training. Right panel showing the progressively increased running distance over time in parallel with the increase in treadmill speed across the 6-week training period. **D** Bar graph indicating the designed progressively increased weight load during the ladder climbing intervention. Ladder climbing weight load was progressively increased from 50% to 60% of body weight across the 6-week intervention. **E** Number of ladder climbs completed during the 12 ladder climbing physical activity sessions, shown separately for individual mice. **F** Body weight changes in the CT-group and PA-group between the post-test time point to the pre-test at13–14 months of age. No significant change in body weight was observed in the CT-group, whereas the PA-group showed a significant reduction in body weight following the 6-week forced treadmill running and ladder climbing physical activity intervention. Data are presented as mean ± SEM with dots representing individual mice (Blue, male; red, female). Statistical significance was determined using two-tailed Wilcoxon matched-pairs signed rank tests. ns, not significant; *, *p* < 0.05

Twenty male and female 3xTg-AD mice were assessed for the baseline cognitive performance to catch age-associated cognition declines with a series of behavior tests at 9–11 months old (Pre-Test 1), 11–12 months old (Pre-Test 2), and 13–14 months old of age (Pre-Test 3), respectively (Fig. 1A). Three cognitive behavior tests were performed, including the novel object recognition (NOR) test, the Y-maze test, and the social behavior test. The NOR test was utilized to assess recognition memory, capitalizing on the innate tendency of rodents to preferentially explore novel objects over familiar ones, an ability largely dependent on hippocampal and cortical integrity [35]. The Y-maze test was conducted to evaluate spatial working memory, as it relies on the rodent’s natural inclination to explore unfamiliar environments, thereby probing both hippocampal and prefrontal cortical function [36]. The social behavior test was employed to assess sociability and social novelty preference, an innate ability also heavily dependent on hippocampal and cortical integrity [37], providing important insights into social cognition, a domain commonly impaired in AD [38].

After these baseline behavior pre-tests, mice were randomly assigned into two experimental groups: forced treadmill running and ladder climbing physical activity group (*n* = 10, referred as the PA-group) and a sedentary control group (*n* = 10, referred as the CT-group). We chose to perform the forced treadmill running and ladder climbing physical activity intervention no later than 13–14 months of age, because the mortality of male and female 3xTg-AD mice was reported to start at 15 months of age [39, 40]. The PA-group mice underwent a six-week long physical activity procedure, including forced treadmill running physical activity (Fig. 1B and C) and ladder climbing physical activity (Fig. 1B, D and E), while the CT-group mice were maintained with a routine husbandry care. Following the six-week forced treadmill running and ladder climbing physical activity, post-tests were performed to assess cognitive functions in PA-group and compared to CT-group. We observed that the six-week forced treadmill running and ladder climbing physical activity procedure caused a statistically significant reduction in body weight in the PA-group mice (*p* < 0.05, Wilcoxon test, Fig. 1F), primarily driven by males (before and after PA for males: 37.4 ± 1.0 and 32.1 ± 0.5, *p* = 0.06; before and after PA for females: 26.4 ± 0.8 gram and 26.3 ± 0.8 gram, *p* = 0.81, Wilcoxon test).

### Forced treadmill running and ladder climbing physical activity procedure improved cognitive performances in aged 3xTg-AD mice

We compared cognitive performances of PA-group and CT-group mice with the NOR, Y-maze, and social behavior tests during pre-tests and post-test. NOR test has two stages, familiarization and testing stages. Normal NOR performance is characterized by the mouse spending significantly more time exploring the novel object during the testing stage. CT-group displayed a normal NOR performance at 9–10 months of age, and became abnormal at 11–12 months of age. PA-group displayed abnormal NOR at all pre-tests, since 9–10 months of age (Supplemental Figure S1). Following the six-week forced treadmill running and ladder climbing physical activity intervention, PA-group mice restored their preference for novel object while CT-group remained abnormality during the testing stage.

**Fig. 2:**
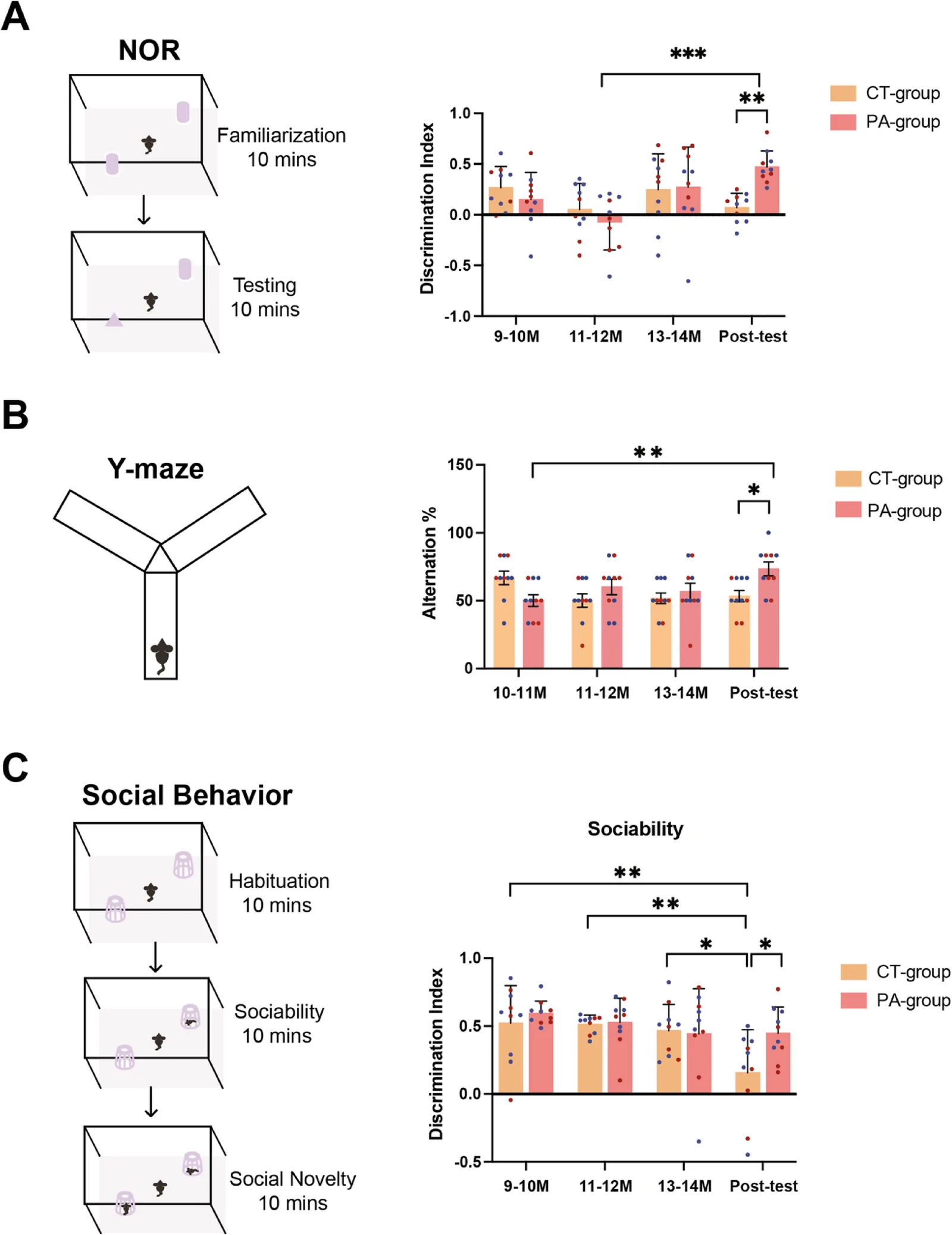
Effects of forced treadmill running and ladder climbing physical activity on recognition memory, spatial working memory, and sociability in aged 3xTg-AD mice. **A** Schematic representation of the NOR test and quantification of the discrimination index. The discrimination index was measured at 9–10, 11–12, and 13–14 months of age before the intervention and at the post-test time point. Following the 6-week intervention, the PA-group exhibited a significantly higher discrimination index compared with the CT-group, and a significant difference between the PA-group at post-test and the 11-month pre-test time point, indicating an improved recognition memory. **B** Schematic representation of the Y-maze test and quantification of spontaneous alternation performance. Spontaneous alternation percentage was assessed at 10–11, 11–12, and 13–14 months of age before the intervention and again at the post-test time point following the 6-week intervention. The PA-group showed improved alternation performance at the post-test compared with the CT-group, and a significant difference between the PA-group at post-test and pre-test at the 10–11 months of age time point, suggesting enhanced spatial working memory after forced treadmill running and ladder climbing physical activity. **C** Schematic representation of the social behavior test and quantification of the discrimination index of sociability during the sociability stage. Sociability performance was evaluated at 9–10, 11–12, and 13–14 months of age before the intervention and at the post-test time point. The PA-group showed preserved sociability at the post-test, whereas the CT-group exhibited impaired sociability at the same time point. Data are presented as mean ± SEM with dots representing individual mice (Blue, male; red, female). Statistical significance was determined using two-way repeated-measures ANOVA followed by Sidak’s multiple comparisons test. CT-group, sedentary control group; PA-group, forced treadmill running and ladder climbing physical activity group; NOR, novel object recognition. *p < 0.05, ***p < 0.001

In order to quantifiably compare NOR performance between CT- and PA- groups across pre-tests and post-test, discrimination index (DI) was used to calculate differences in exploration time towards novel and familiar objects during the testing stage of individual tests (Fig. 2A). Following the six-week forced treadmill running and ladder climbing physical activity intervention, DI was significantly increased in the PA-group, compared to CT-group during the post-test (*p* < 0.01, two-way Analysis of Variance, or two-way ANOVA and Sidak’s multiple comparisons test). In addition, the PA-group showed a significant increase in DI compared with the pre-test at 11–12 months of age (*p* < 0.001, two-way ANOVA and Sidak’s multiple comparisons test). These results indicated that forced treadmill running and ladder climbing physical activity procedure improved object recognition memory in aged 3xTg-AD mice.

In the Y-maze test, spontaneous alternation was quantified for individual mice. PA-group 3xTg-AD mice displayed ~ 50% spontaneous alternation rate during pre-tests since their 10–11 months of age, while CT-group mice displayed ~ 50% spontaneous alternation rate during pre-tests after their 11–12 months of age (Supplemental Figure S2), indicating a comparable disrupted pre-intervention performance on Y-maze test. Following the six-week forced treadmill running and ladder climbing physical activity intervention, the PA-group displayed a significantly increased alternation rate, compared to the CT-group during the post-test (*p* < 0.05, two-way ANOVA and Sidak’s multiple comparisons test) and its pre-test at 10–11 months of age (*p* < 0.01, two-way ANOVA and Sidak’s multiple comparisons test, Fig. 2B), suggesting that forced treadmill running and ladder climbing physical activity procedure improved spatial working memory in aged 3xTg-AD mice.

Social behavior test has three stages: habituation (H), sociability (SB), and social novelty (SN) stages. Normal sociability is characterized by the mouse spending significantly more time exploring the social target during the SB stage. Normal social novelty is characterized by the mouse spending significantly more time exploring the novel social target than the familiar social target during the SN stage. Both CT-group and PA-group displayed normal sociability during the SB stage across all pre-tests (Supplemental Figure S3). Both CT-group and PA-group displayed normal social novelty during the SN stage of most pre-tests, except for CT-group at 9–10 months of age and PA-group at 13–14 months of age (Supplemental Figure S3). Following the six-week forced treadmill running and ladder climbing physical activity intervention, PA-group mice preserved their sociability while CT-group lost sociability in the SB stage of the post-test, and preference for social novelty was lost for both CT-group and PA-group in the SN stage of the post-test (Supplemental Figure S3).

In order to quantifiably compare social performance between CT- and PA- groups across pre-tests and post-test, DI was used to calculate differences in exploration time towards social target and non-social target during the SB stage for sociability comparisons, and differences in exploration time towards novel social target and familiar social target during the SN stage for social novelty comparisons of individual tests, respectively. For sociability comparisons, both CT-group and PA-group 3xTg-AD mice displayed stable DI baselines across pretests between 9 and 14 months of ages. However, the CT-group showed a significant decreased DI relative to the PA-group, as well as to its own baseline during the post-test (*p* < 0.05 and *p* < 0.01, two-way ANOVA and Sidak’s multiple comparisons test, Fig. 2C) at their 16–17 months of age. In contrast, the PA-group maintained a similar DI at the post-test compared to its baseline, indicating a preserved sociability by physical activity intervention. Both PA-group and CT-group displayed a similar DI for social novelty across pretests at their 9–14 months old age, and physical activity did not improve DI for social novelty (Supplemental Figures S4).

We therefore concluded that six-week forced treadmill running and ladder climbing physical activity procedure attenuated age-associated cognitive declines in aged 3xTg-AD mice, evidenced by the preserved spatial working memory, object recognition memory, and sociability.

### Forced treadmill running and ladder climbing physical activity procedure reduced protein aggregates and gliosis in aged 3xTg-AD mice

**Fig. 3:**
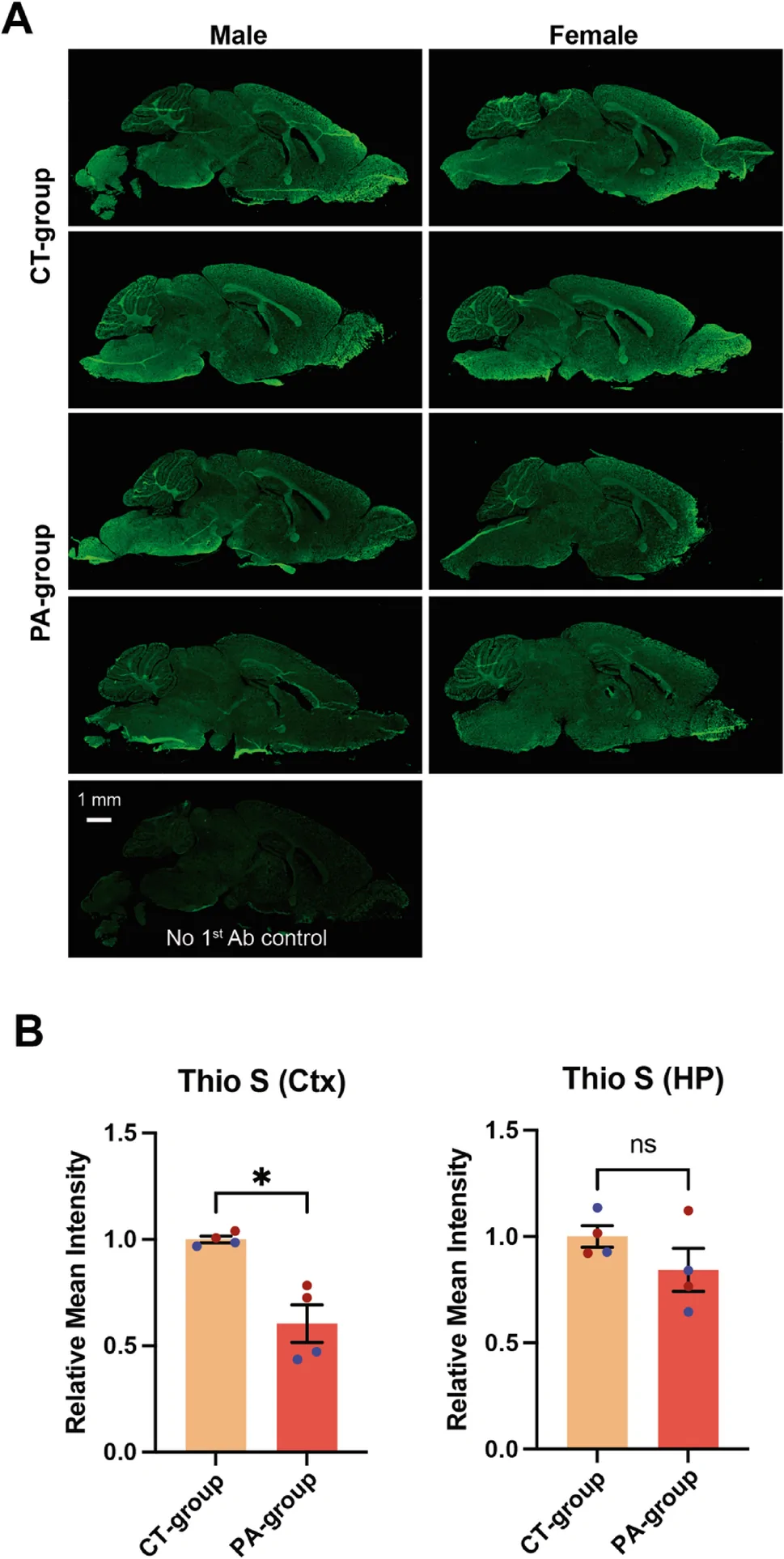
Forced treadmill running and ladder climbing physical activity reduces cortical Thioflavin S signal in aged 3xTg-AD mice. **A** Representative sagittal brain sections showing Thioflavin S staining in male and female mice from the sedentary control group and forced treadmill running and ladder climbing physical activity group. Thioflavin S–positive signal was detected throughout the brain sections and was used to assess misfolded protein aggregates with β-sheet structure. Representative images from both sexes are shown for each experimental group. A no-primary-antibody control section is included to demonstrate background fluorescence. Scale bar = 1 mm. **B** Quantification of relative Thioflavin S mean fluorescence intensity in the cortex and hippocampus. In the cortex, the physical activity group showed a significant reduction in Thioflavin S signal compared with the sedentary control group. In the hippocampus, Thioflavin S mean intensity showed no significant difference between groups. Data are presented as mean ± SEM with dots representing individual mice (Blue, male; red, female). Statistical significance was determined using two-tailed unpaired t-test with Welch’s correction. CT-group, sedentary control group; PA-group, forced treadmill running and ladder climbing physical activity group; Ctx, cortex; HP, hippocampus; Thio S, Thioflavin S; ns, not significant; *p < 0.05

To evaluate the impact of forced treadmill running and ladder climbing physical activity procedure on pathology, we first quantified Aβ and pTau pathology in the hippocampus and frontal cortex. Hippocampus is important for spatial memory and frontal cortex is largely involved in recognition memory and social cognition [37]. Using Aβ specific antibodies, classic extracellular amyloid plaques were only detected in the striatum of this cohort of 3xTg-AD mice. In the frontal cortex and hippocampus, amyloid aggregates were mainly detected as intracellular amyloid immunoreactivity (Supplemental Figures S5A), as Odde et al. previously reported in 2003 [41]. We performed Thio S staining to compare misfolded protein aggregates in the frontal cortical and hippocampal regions of CT-group and PA-group mice (Fig. 3A). Thio S is a fluorescent dye widely used to visualize misfolded protein aggregates with β-sheet structure property, such as Aβ plaque and tau tangles [42–44]. Co-staining of Thio S and Aβ or pTau specific antibodies confirmed the colocalization of Thio S staining with the intracellular amyloid and pTau immunoreactivity in both the cortex and hippocampus (Supplemental Figures S5B). We also confirmed that Thio S did not colocalize with glial markers, such as Glial Fibrillary Acidic Protein (GFAP) and Ionized Calcium-Binding Adapter Molecule 1(IBA1) in the cortex and hippocampus (Supplemental Figures S5C). These observations suggested that Thio S staining picked up intraneuronal pathology containing both amyloid and pTau immunoreactivity.

Quantitative analysis of Thio S fluorescence intensity revealed a significant reduction in the cortex of PA-group mice compared to CT-group (*p* < 0.01, unpaired t-test). In contrast, no statistically significant difference was detected in the hippocampus between the two groups (Fig. 3B). To our surprise, we did not detect significant differences between the PA-group and CT-group when using Aβ or pTau specific antibodies (Supplemental Figures S6, S7). We interpret these results as that our forced treadmill running and ladder climbing physical activity procedure reduced intracellular aggregates likely including both Aβ and pTau while the protective effects on single species of aggregates varied in different mice.

**Fig. 4:**
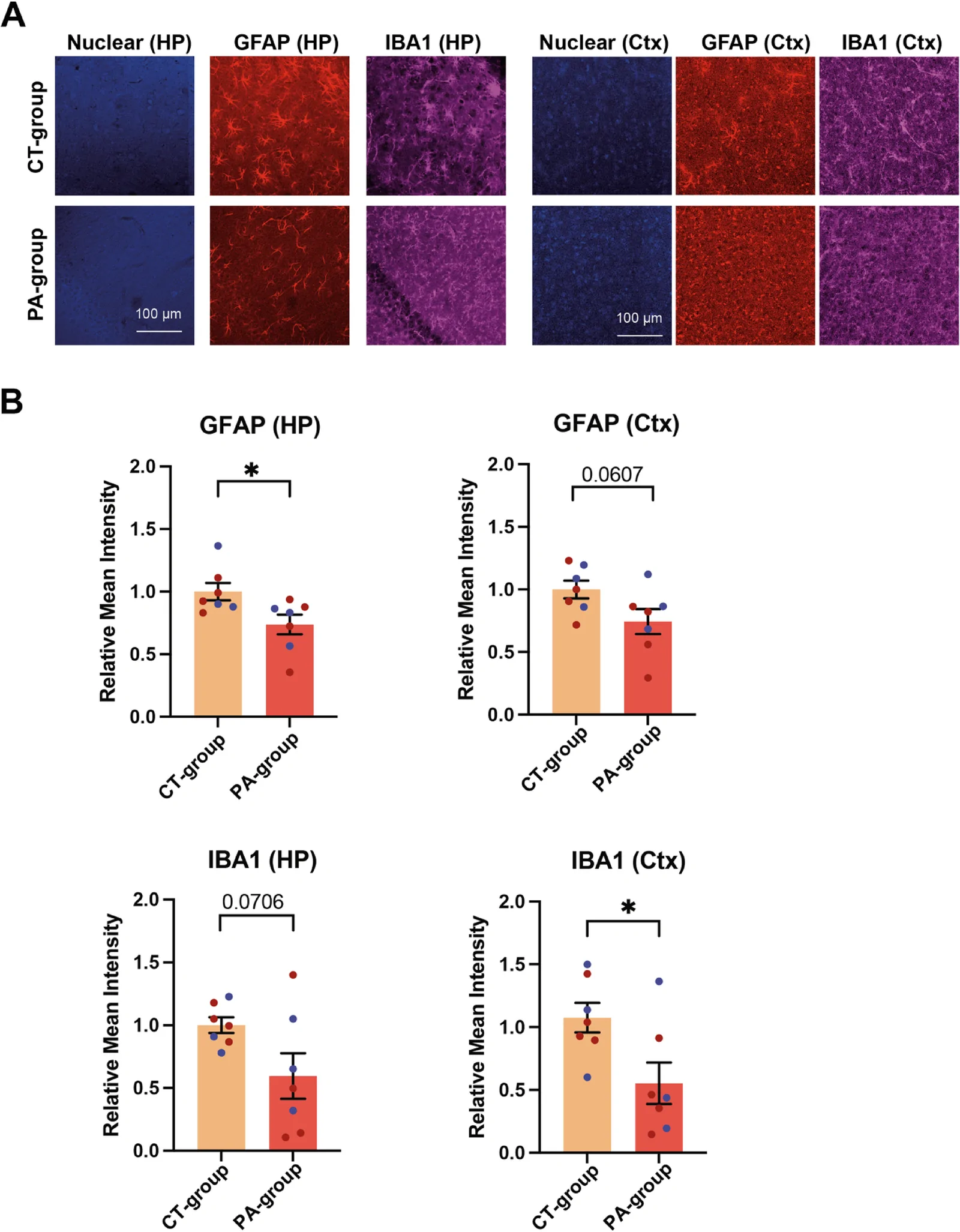
Forced treadmill running and ladder climbing physical activity reduces glial activation in the hippocampus and cortex of aged 3xTg-AD mice. **A** Representative fluorescence images showing IBA1 and GFAP staining, from the cortex and hippocampus of CT-group and PA-group of 3xTg-AD mice. IBA1 was used to label microglia, GFAP was used to label astrocytes. Scale bar = 100 μm. **B** Quantification of relative mean fluorescence intensity for GFAP and IBA1 in the hippocampus and cortex. In the hippocampus, the physical activity group showed significantly reduced GFAP intensity compared with the sedentary control group, while IBA1 intensity showed a trend toward reduction. In the cortex, IBA1 intensity was significantly reduced in the physical activity group compared with the sedentary control group, while GFAP intensity showed a trend toward reduction. Data are presented as mean ± SEM with dots representing individual mice (Blue, male; red, female). Statistical significance was determined using two-tailed unpaired t-test with Welch’s correction. CT-group, sedentary control group; PA-group, forced treadmill running and ladder climbing physical activity group; HP, hippocampus; Ctx, cortex; IBA1, ionized calcium-binding adaptor molecule 1; GFAP, glial fibrillary acidic protein; Thio S, Thioflavin S. *p < 0.05

To evaluate whether 6-week forced treadmill running and ladder climbing physical activity procedure modulated reactive gliosis responses in the cortex and hippocampus, astrocytic and microglial activation were assessed using GFAP and IBA1 immunostaining, respectively (Fig. 4). Quantitative analysis (Fig. 4B) revealed that GFAP levels in the hippocampus were significantly reduced in the PA-group relative to the CT-group (*p* < 0.05, Unpaired Welch t-test), suggesting a reduction in reactive astrocytes following the forced treadmill running and ladder climbing physical activity intervention. GFAP immunoreactivity displayed a downward trend in the cortex of PA-group mice compared with CT-group (*p* = 0.0607, Unpaired Welch t-test).

Quantitative analysis also revealed a significant reduction in IBA1 immunoreactivity in the cortex of the PA-group compared with the CT-group (*p* < 0.05, Unpaired Welch t-test), indicating decreased reactive microglia following the forced treadmill running and ladder climbing physical activity intervention. In the hippocampus, IBA1 expression also showed a decreasing trend in the PA-group, although this difference did not reach statistical significance (*p* = 0.0706, Unpaired Welch t-test). Collectively, these findings suggested that forced treadmill running and ladder climbing physical activity attenuated reactive gliosis in aged 3xTg-AD mice, particularly by reducing microglial activation in the cortex and astrocytic reactivity in the hippocampus, indicating a potential anti-inflammatory effect of the forced treadmill running and ladder climbing physical activity procedure on the AD brain.

### Forced treadmill running and ladder climbing physical activity modulated the inhibitory circuitry in aged 3xTg-AD mice

**Fig. 5:**
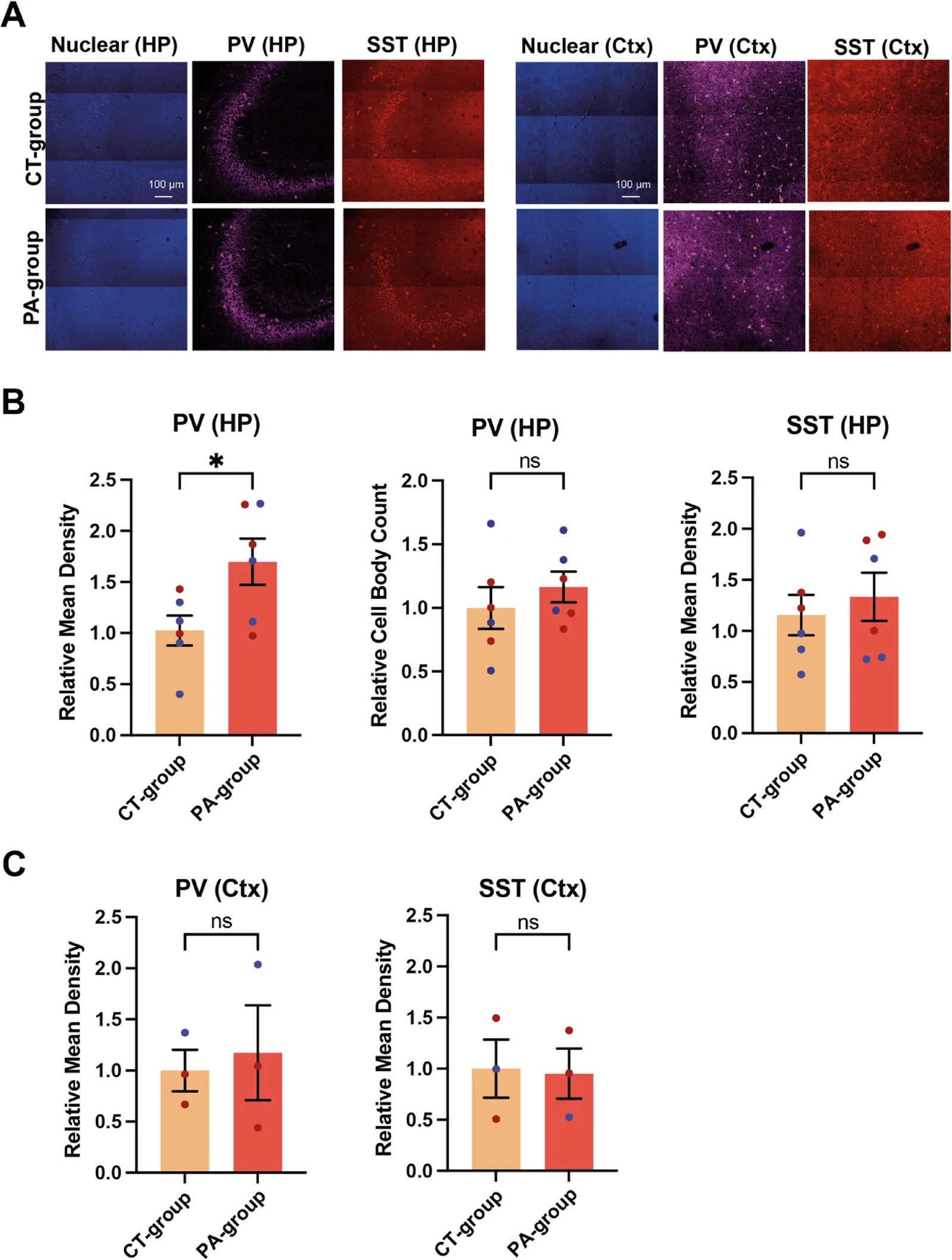
Forced treadmill running and ladder climbing physical activity increases hippocampal parvalbumin-positive immunostaining particles in aged 3xTg-AD mice. **A** Representative hippocampal and cortical fluorescence images showing parvalbumin (PV) and somatostatin (SST) immunoreactivity in sedentary control mice (CT-group) and forced treadmill running and ladder climbing physical activity mice (PA-group). Scale bars are 100 μm. **B** Bar graphs showing quantifications of relative immunostaining particle density for parvalbumin-positive particles in the hippocampus *via* QuPath automatic counting (left), relative parvalbumin-positive cell bodies in the hippocampus *via* manual counting (middle), and relative immunostaining particle density for somatostatin-positive particles in the hippocampus *via* QuPath automatic counting. **C** Quantitative analysis of relative mean immunostaining particle density for parvalbumin- and somatostatin-positive fluorescence particles in the cortex *via* QuPath automatic counting. Data are presented as mean ± SEM with dots representing individual mice (Blue, male; red, female). Statistical significance was determined using two-tailed unpaired t-test with Welch’s correction. CT-group, sedentary control group; PA-group, forced treadmill running and ladder climbing physical activity group; HP, hippocampus; Ctx, cortex; PV, parvalbumin; SST, somatostatin; ns, not significant; *p < 0.05

Loss of somatostatin (SST)-positive inhibitory interneurons [7–13] and loss of parvalbumin (PV)-positive inhibitory interneurons [5–7, 45] have been reported in various brain regions of AD patients and AD mouse models. To investigate whether the forced treadmill running and ladder climbing physical activity procedure affected inhibitory circuitry, immunostaining of inhibitory neuron markers were performed and PV-positive or SST-positive immunostaining particles in the hippocampus and cortex of the PA-group and CT-group were automatically counted and compared *via* QuPath, with a combined threshold for particle intensity above 7000 and particle size of 25 ~ 5000 µm^2^ (Fig. 5A). Automatic analysis revealed a significant increase in PV-positive immunostaining particle density of the PA-group compared with the CT-group in the hippocampus (*p* < 0.05, Unpaired Welch t-test), but not in other brain regions (Fig. 5B and C). This effect appeared to be specific, as no significant changes were observed with SST-positive immunostaining particle density. We also manually counted PV-positive cell bodies and found that manually identified PV-positive cell bodies were not significantly different in the hippocampus between the PA-group and CT-group. This discrepancy might be due to that manual counting only identified big obvious cell bodies, whereas automatic particle counting might include the PV-positive particles in the neuronal processes or segments of neurons that appear to be smaller.

**Fig. 6:**
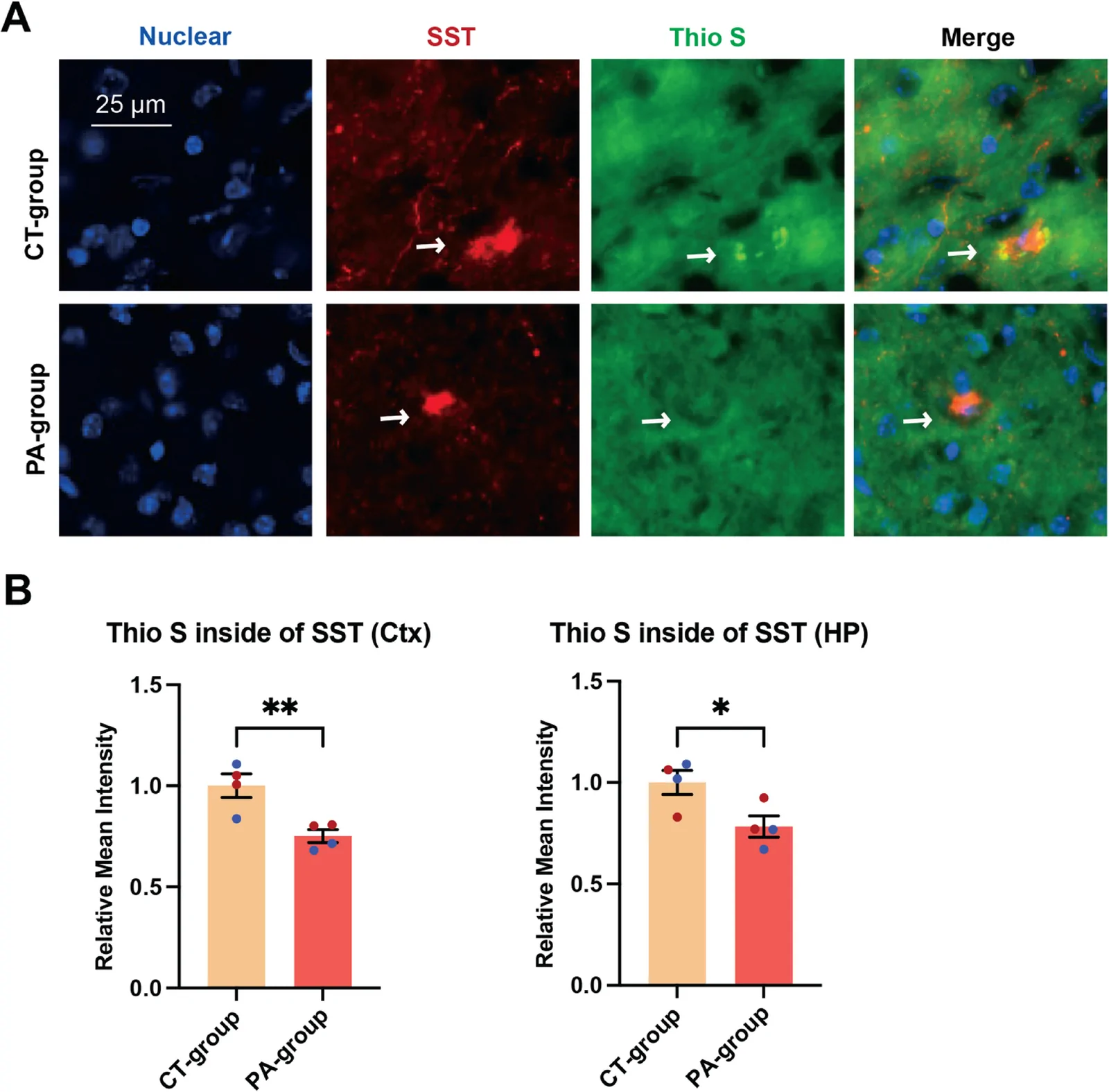
Forced treadmill running and ladder climbing physical activity decreases Thioflavin S accumulation in SST-positive interneurons of aged 3xTg-AD mice. **A** Representative fluorescence images of brain sections stained for nuclei, SST, and Thioflavin S in sedentary control and forced treadmill running and ladder climbing physical activity groups. Merged images illustrate the distribution of Thioflavin S signal relative to SST-positive interneurons. White arrows indicate regions with overlapping SST-positive inhibitory interneurons and Thioflavin S–positive signal. Scale bar = 25 μm. **B** Quantitative analysis of relative Thioflavin S mean fluorescence intensity within SST-positive interneurons in the cortex and hippocampus. Forced treadmill running and ladder climbing physical activity significantly reduced Thioflavin S intensity within SST-positive interneurons in both the cortex and hippocampus compared with sedentary controls. Data are presented as mean ± SEM with dots representing individual mice (Blue, male; red, female). Statistical significance was determined using two-tailed unpaired t-test with Welch’s correction. CT-group, sedentary control group; PA-group, forced treadmill running and ladder climbing physical activity group; Ctx, cortex; HP, hippocampus; SST, somatostatin; Thio S, Thioflavin S; *p < 0.05, **p < 0.01

To further investigate whether the forced treadmill running and ladder climbing physical activity influences misfolded protein aggregates within inhibitory interneurons, Thio S staining was co-stained with markers of inhibitory interneurons (Fig. 6A). Quantitative analysis revealed that the relative intensity of Thio S within SST-positive inhibitory interneurons was significantly reduced in the PA-group compared with the CT-group, in both the cortex and hippocampus (*p* < 0.01, Unpaired t-test) (Fig. 6B). In contrast, Thio S staining within PV-positive inhibitory interneurons was relatively weak, and the intensity was similar between PA-group and CT-group in both the cortex and hippocampus (Supplemental Figures S8). These findings indicate that forced treadmill running and ladder climbing physical activity reduces Thio S positive neuropathology specifically within SST-positive inhibitory interneurons, suggesting that forced treadmill running and ladder climbing physical activity procedure may alleviate misfolded protein aggregates within specific neuronal subpopulations in aged 3xTg-AD mice.

## Discussion

In the present study, we demonstrated that aged 3xTg-AD mice (13–14 months of age) that underwent a 6-week forced treadmill running and ladder climbing physical activity program modulated age-associated cognitive declines in spatial working memory, object recognition memory, and sociability. These behavioral improvements were accompanied by reduced cortical misfolded protein aggregates, attenuated glial activation, and notably, decreased Thio S labeled neuropathology within SST-positive interneurons in both the cortex and hippocampus. To our knowledge, this work represents one of the first demonstrations of the benefits of forced treadmill running and ladder climbing physical activity procedure on aged 3xTg-AD mice. Additionally, our results suggest that forced treadmill running and ladder climbing physical activity alleviates Thio S positive pathology specifically at the cellular level (i.e., in a particular subtype of inhibitory interneurons), rather than indistinguishably at the global whole-tissue level. We therefore propose that the forced treadmill running and ladder climbing physical activity procedure, as short as the 6-week duration, is capable to provide a multi-level intervention by targeting protein aggregates and gliosis, thereby offering a comprehensive non-pharmacological intervention strategy to mitigate cognitive decline in AD.

### Forced treadmill running and ladder climbing physical activity improved cognitive functions in aged 3xTg-AD mice

It is well documented that involuntary aerobic physical activity such as forced treadmill running and voluntary aerobic physical activity such as voluntary wheel running elicit potent protective effects to slow down the disease progression in various AD rodent models [20, 26]. For instance, 12-week forced treadmill running on 3×Tg-AD mice improved their spatial working memory [21, 22, 25]; and 6-week forced treadmill running on the presenilin-2 mutant AD mice, or 4-week forced treadmill running on rats injected with Aβ42, increased their preference in exploring a novel object [46, 47]. However, a major drawback in these studies is that most of them performed involuntary aerobic physical activity trainings on young animals (2–4 months old). Additionally, it is worth noting that forced treadmill running induced elevated anxiety and corticosterone, while voluntary wheel runners showed no such stress response [48, 49].

Compared to aerobic physical activity studies, fewer efforts have been made to study the effects of resistance physical activity on AD rodent models. Short-term (4 weeks) resistance physical activity was reported to have beneficial effects on hyperlocomotion in 6–7 months old APP/PS1 mice [50, 51], and reversed memory deficits in 7–8 months old female APP/PS1 mice [52]; while long-term (4 months) resistance physical activity rescued different types of memory in 6 months old female and male APP/PS1 mice [53].

To our knowledge, the present study is the first forced treadmill running and ladder climbing physical activity performed on aged 3xTg-AD mice (13–14 months of age). Consistent with previous studies, we observed significant beneficial effects of spatial memory and recognition memory on these aged 3xTg-AD mice, demonstrated by the enhanced Y-maze and NOR performances with just 6 weeks of forced treadmill running and ladder climbing physical activity.

Besides deficits on learning and memory, 3xTg-AD mice also displayed behavioral and psychological symptoms of dementia (BPSD), such as anxiety and depression-like behaviors [54, 55]. It has been reported that long-term (3 to 6 months) voluntary wheel running ameliorated anxiety, apathy, and startle responses in 7 months old and 15 months old 3xTg-AD mice [56, 57]. Here we demonstrated that the 6-week forced treadmill running and ladder climbing physical activity procedure modulated age-associated sociability decline in aged 3xTg-AD mice.

Alongside the behavioral changes, there was a significant reduction in body weight observed in the PA-group following 6-week forced treadmill running and ladder climbing physical activity, consistent with the previous reports on aged 3xTg-AD mice (12–15 months of age), with improved body fitness following either the 4-month combined voluntary wheel running and forced treadmill running procedure or the 3-month voluntary wheel running procedure [57, 58].

### Forced treadmill running and ladder climbing physical activity reduced cortical misfolded protein aggregates and reactive gliosis in aged 3xTg-AD mice

There is a growing body of evidence showing that both voluntary and involuntary aerobic exercise trainings on various AD mouse models reduces cortical and/or hippocampal Aβ plaque burden [22, 57–62], reduces hippocampal pTau levels [57, 60], suppresses gliosis and neuroinflammatory cascades [22, 62–67], restores synaptic integrity [25, 47, 66, 68, 69], increases mitochondrial function [22, 58], and promotes hippocampal neurogenesis [22]. Studies for the resistance exercise trainings on Aβ burden have mostly yielded beneficial results [50–52] with only one exception [53].

Our results demonstrated that 6-week forced treadmill running and ladder climbing physical activity significantly reduced Thio S labeled misfolded protein aggregates in the cortex, but not the hippocampus of aged 3xTg-AD mice. We also observed significant reductions in hippocampal GFAP and cortical IBA1, and trends of reduction in hippocampal IBA1 (*p* = 0.0706) and cortical GFAP (*p* = 0.0607). We did not observe any significant effects on hippocampal or cortical pTau levels. The differences between our findings and those previously reported benefits may relate to differences in physical activity protocol (e.g., physical activity type, intensity, and duration), the age when physical activity was initiated, and/or the specific transgenic model used. Thomas et al. (2020) demonstrated that the anti-amyloid effects of physical activity in Tg2576 mice were intensity dependent. High-intensity training reduced plaque load in both cortex and hippocampus, whereas low-intensity training had a more limited effect [70]. It is worth noting that the durations of most previously reported effective involuntary treadmill running and voluntary wheel running were typically lasted from 10 weeks to 5 months [22, 59, 60, 64, 65]. Compared to these previous studies, the duration of our forced treadmill running and ladder climbing physical activity procedure falls at the short-term range. Another limitation in our study was the lack of physiological measurements (e.g., VO_2_ max, the maximal oxygen consumption or time-to-exhaustion for aerobic fitness, and grip strength or muscle cross-sectional area for resistance adaptation) to demonstrate that our 6-week forced treadmill running and ladder climbing physical activity protocol produced the desired systemic outcomes. Nevertheless, improvements of cognitive performance occurred even without robust anti-amyloid or anti-pTau effects. Such cognitive resilience has been previously reported [71].

### Forced treadmill running and ladder climbing physical activity potentially modulated vulnerable inhibitory circuitry in aged 3xTg-AD mice

Although inhibitory interneurons only occupy ~ 20% of total cortical neurons, their circuitry plays a critical role in maintaining the excitation-inhibition balance and stabilizing neural networks in the healthy brain. Among several major subtypes of inhibitory interneurons, PV-positive and SST-positive inhibitory interneurons have been mostly studied and linked to AD. Loss of PV-positive interneurons, decreased PV expression, and dysfunctional PV-positive interneurons have been reported across multiple AD transgenic mouse lines and human AD patients [5–7]. It has been reported ~ 52% loss of PV-positive interneurons in the hippocampus of 18 months old 3xTg-AD mice [45].

SST-positive interneurons are perhaps among the earliest detected cell types with a severe loss and the most vulnerable neuronal populations in AD [7–9, 11, 12]. Ramos et al. (2006) reported a 50–60% reduction in SST-positive interneurons in the hippocampus of PS1×APP mice as early as 6 months of age, preceding pyramidal cell loss [11]. This selective vulnerability of SST-positive interneurons appears to be consistent across AD mouse models and human AD patients [7–10, 12, 13]. In contrast, cognitive resilient individuals exhibited a better survival of SST-positive inhibitory interneurons in the cortex [72]. Besides the vulnerability of SST-positive interneurons towards Aβ toxicity, a bidirectional relationship between SST and Aβ in AD has been suggested, as SST may play a regulatory role for Aβ clearance. SST regulates Aβ42 metabolism and genetic SST deficiency increases hippocampal Aβ42 [73]; SST also interferes with Aβ fibrillization, being identified as the most selectively enriched binder to oligomeric Aβ42 in human frontal lobe tissues [74].

Neuron loss, reduced expression of PV and SST, or dysfunction in PV-positive and SST-positive inhibitory interneurons often leads to excitation-inhibition imbalance and associates with network hyperexcitability [15]. AD patients displayed increased incidents of seizures at early stages [16, 75–78], presumably caused by the dysfunctional inhibitory circuitry. It has been reported that 16 weeks forced treadmill physical activity on 6 months old 3xTg-AD mice leads to prevention of neuronal hyperexcitability in the medial entorhinal cortex, a region critical for spatial memory that is affected early in AD [21].

Importantly, our results showed that 6-week forced treadmill running and ladder climbing physical activity procedure significantly reduced Thio S positive neuropathology within the SST-positive inhibitory interneurons in both the cortex and hippocampus of aged 3xTg-AD mice. Most previous studies assessed Aβ reduction only globally at the whole-tissue level without distinguishing cell-type-specific effects. To our knowledge, the present study is the first report to demonstrate the effects of physical activity on misfolded protein aggregates at levels of specific interneuron subtypes, adding an important new dimension to the current understanding about neuroprotective mechanisms of physical activity. Whether the 6-week forced treadmill running and ladder climbing physical activity procedure achieves this specifically within SST neurons by enhancing clearance for intracellular Aβ and/or tau tangle, by reducing Aβ or pTau production, or indirectly through systemic anti-inflammatory or neurotrophic effects remains to be determined.

Given the critical roles of PV-positive and SST-positive inhibitory interneurons in maintaining excitation-inhibition balance and supporting cortical and hippocampal oscillations essential for memory [79, 80], our observations of selective reduction of Thio S labeled neuropathology within SST-positive interneurons may represent a particularly meaningful mechanism by which the forced treadmill running and ladder climbing physical activity procedure preserves cognitive function.

### Limitations of this study

We would like to acknowledge several important limitations in the present study. (1) Lack of a comparable dataset from age-matched wild type (WT) controls. Future experiments with age-matched WT controls will help to distinguish effects on age-related or AD-related cognitive declines, gliosis, and neuropathology. (2) Lack of comparable datasets from treadmill running-only and ladder climbing-only training groups from the age-matched 3xTg-AD mice. Future experiments with such controls will help to determine if combination of different types of physical activity would achieve synergistic or additive effects. (3) Lack of physiological markers for aerobic fitness and resistance adaptation to conform the designed training protocol is truly functioned as targeted trainings. (4) Lack of body weight-matched control group to distinguish the effects of exercise from those of reduced body weight. (5) There was not enough power to assess potential sex-difference due to the small n numbers.

In summary, we demonstrated several beneficial effects of a six-week forced treadmill running and ladder climbing physical activity procedure on 13–14 months old 3xTg-AD mice, with reduced body weight, improved cognition, reduced cortical misfolded protein aggregates, reduced gliosis in the hippocampus and cortex, and reduced Thio S labeled neuropathology within SST-positive inhibitory interneurons in the hippocampus and cortex. Our results support physical activity as a promising non-pharmacological intervention for modulating disease-related pathology and preserving cognition in AD.

## Methods

### Animals

The experiments described here were approved by the Institutional Animal Care and Use Committee (IACUC) at the University of Wyoming and were conducted in accordance with NIH guidelines for the care and use of laboratory animals. Homozygous 3xTg-AD mice were purchased from The Jackson Laboratory (strain #003930) and maintained at the animal facility of University of Wyoming. All mice were group-housed under artificial light (12 h light/dark cycle) at 22 ± 2 °C with ad libitum access to food and water.

### Experimental design and timeline

Male and female 3xTg-AD mice were used in our study. Three baseline behavioral assessments were conducted prior to the intervention period, at 9–11 months,11–12 months, and 13–14 months of ages, referred as Pre-Tests 1 to 3. Pre-tests included the novel object recognition (NOR) test, the Y-maze test, and the social behavior test to evaluate recognition memory, spatial working memory, and sociability, respectively. Following these baseline tests, mice were randomly assigned into two subgroups: forced treadmill running and ladder climbing physical activity and sedentary control group (i.e., PA-group the CT-group). The PA-group mice underwent a 6-week long forced treadmill running and ladder climbing physical activity procedure, while the CT-group mice were maintained with a routine husbandry care. Following the 6-week forced treadmill running and ladder climbing physical activity intervention protocol, a similar set of behavior tests was performed at 16–17 months of age to evaluate physical activity induced changes in cognitive performance. Brain tissues were then collected for immunostaining analyses to explore underlying molecular and cellular mechanisms.

All behavior tests and physical activity procedures occurred at room temperature during the daytime, specifically from 10:00 AM to 6:00 PM.

### Forced treadmill running and ladder climbing physical activity procedure

Forced treadmill running physical activity: A motorized treadmill with a digitally controlled unit offering high repeatability (AT-5MR, Shanghai TOW Intelligent Technology Co., Ltd) was used in this study. Treadmill running physical activity underwent a one-week habituation (0.1 m/s) period to acclimate the treadmill environment. Throughout the habituation period, mice were gently trained to adapt to running on a rotating belt of the motorized treadmill. The forced treadmill running training program lasted for six weeks, with sessions held three days per week (Fig. 1C). For the first two weeks, mice run at a speed of 0.2 m per second for 40 min per session. In the third and fourth weeks, the speed increased to 0.25 m per second for the same duration. During the fifth and sixth weeks, the speed was further increased to 0.3 m per second, maintaining the 40-minute duration. Each training session also included a 5-minute warm-up at a speed of 0.1 m per second and a 5-minute cool-down at the same speed.

Ladder climbing physical activity: Ladder climbing physical activity was accomplished utilizing a 1-meter long ladder with 2-centimeter grid steps and inclined at 85° (TOW-PT-VMR85, Shanghai TOW Intelligent Technology Co., Ltd). Each mouse was attached with weight load rings (Jinan Yiyan technology development co., LTD) on the tail during ladder climbing training. The ladder climbing physical activity program lasted for six weeks, with sessions held two times per week (Fig. 1D). For the first two weeks, the mice performed climbs bearing 50% of their body weight (weighed on the first training day). In the third and fourth weeks, the weight bearing increased to 55% of body weight. For the final two weeks, the mice climbed with 60% of body weight. During each physical activity session, with a 1-minute rest interval between each climb until fatigue (e.g., failure to continue climbing; inability to maintain coordinated movement; repeated unsuccessful attempts). Only gentle tail touching was used to motivate the mice to climb continuously.

### Y-maze test

The test protocol spanned four consecutive days and was designed to assess spatial working memory. On both Days 1 and 2, mice underwent habituation phases to minimize anxiety-related confounds and familiarize them with the maze environment. During these habituation phases, mice were allowed to freely explore the maze for 10 min without any specific constraints or goals. On Day 3, the forced alternation training phase was conducted with a total of 7 trials per mouse. Each trial began by placing the mouse in a designated “Start” arm, where it remained for 30 s. After this period, the door was opened to allow the mouse to freely choose and enter one of the two goal arms. Upon entering an arm, the door was closed, and the mouse was confined to the selected arm for an additional 30 s. To encourage alternation behavior, if the mouse selected the same goal arm for the third time, the door remained open until the mouse made a correct alternative. Upon correct selection, the mouse was confined to that arm for 30 s.

Day 4 was the alternation testing phase, comprising 8 trials. As before, mice were placed in the “Start” arm, but the confinement period was extended to 90 s to increase retention demands. For Trials 1 to 7, the door was opened, and the mouse was allowed to choose a goal arm; upon selection, it was confined to that arm for 30 s. In Trial 8, the inter-trial confinement in the “Start” arm was shortened to 5 s to check for motivation. This would help to ensure that any observed changes in performance on the spontaneous alternation task were due to changes in memory rather than changes in motivation. The alternation rate was calculated as the percentage of correct alternations relative to the maximum number of alternations possible. A correct alternation was defined as the selection of a different goal arm from that chosen in the immediately preceding trial. The alternation rate was computed as:

$$\begin{aligned} \mathrm{alternation}\;\mathrm{rate}\;&(\%)\;=\;(\mathrm{number}\;\mathrm{of}\;\mathrm{correct}\;\mathrm{alternations}\;\\&/\;\mathrm{maximum}\;\mathrm{possible}\;\mathrm{alternations})\;\times\;100 \end{aligned}$$

with the maximum number of alternations defined as the total number of valid trials minus one.

### Novel Object Recognition (NOR) test

The NOR test was conducted to assess recognition of memory in mice, based on their natural tendency to explore novel objects over familiar ones. Mice were first habituated to the testing environment by being placed in the experimental room for 30 min. This step aimed to reduce novelty-induced anxiety and allow the animals to acclimate their surroundings. Following the habituation, each mouse was placed in a clean open field arena (42 × 42 × 30 cm) containing two identical objects. The mouse was allowed to freely explore the objects for 10 min. After the familiarization stage, the mouse was returned to its holding cage for a 5-minute rest period. The mouse was then reintroduced to the same arena, which now contained one of the previously encountered familiar objects and a novel object with a different shape and texture. During this testing stage, the animal was allowed to explore freely for 10 min. Exploration of time directed toward each object was recorded to calculate the recognition index, which serves as an indicator of recognition memory performance. All sessions were conducted under controlled lighting and noise conditions, and the arenas and objects were thoroughly cleaned between trials to eliminate olfactory cues. The discrimination index (DI) was calculated to assess recognition memory performance as:

$$\mathrm{DI}=({\mathrm T}_-\mathrm{novel}-{\mathrm T}_-\mathrm{familiar})/({\mathrm T}_-\mathrm{novel}+{\mathrm T}_-\mathrm{familiar})$$

where T_novel and T_familiar denote the time spent exploring the novel and familiar objects during the testing stage, respectively.

### Social behavior test

The Social Behavior Test was performed to assess sociability and social novelty preference in mice. The test comprises three sequential stages: habituation stage, sociability stage, and social novelty stage, each lasting 10 min. First, each mouse was placed in the center of the arena and allowed to freely explore two empty wire cages (inverted small metal cup) placed in the two corners. This 10-minute habituation stage allowed the mice to acclimate the testing environment and minimize novelty effects during subsequent phases. Following habituation, one unfamiliar conspecific mouse (Stranger 1) of the same age and sex as the test mouse was placed under one of the wire cages, while the other cage remained empty. During the sociability stage, the test mouse was reintroduced to the center arena and allowed to explore freely for 10 min. The amount of time spent in proximity to each wire cage was recorded to assess the mouse’s preference for social interaction. Immediately following the sociability stage, a second unfamiliar mouse (Stranger 2), also age- and sex-matched, was introduced under the previously empty wire cage, while Stranger 1 remained in place. During the social novelty stage, the test mouse was again allowed access to the arena for 10 min. Time spent interacting with the familiar (Stranger 1) versus novel conspecific (Stranger 2) was recorded to evaluate preference for social novelty. Sociability and social novelty preference were quantified using discrimination indices (DI). The sociability DI was defined as:

$$({\mathrm T}_-\mathrm{stranger}1-{\mathrm T}_-\mathrm{empty})/({\mathrm T}_-\mathrm{stranger}1+{\mathrm T}_-\mathrm{empty})$$

where T_stranger1 and T_empty denote time spent interacting with the unfamiliar mouse and the empty cage during the sociability stage, respectively.

The social novelty DI was defined as:

$$({\mathrm T}_-\mathrm{stranger}2-{\mathrm T}_-\mathrm{stranger}1)/({\mathrm T}_-\mathrm{stranger}2+{\mathrm T}_-\mathrm{stranger}1)$$

where T_stranger2 and T_stranger1 denote time spent interacting with the novel and familiar conspecifics during the social novelty stage, respectively.

### Brain tissue harvesting and immunohistochemistry

Mice brains were harvested after post-test by cardiac perfusion with phosphate buffered saline (PBS) followed by 4% paraformaldehyde (PFA). The brains were then post-fixed overnight in 4% PFA and stored at 4 °C until sectioning. The fixed brains were either sectioned into 70 μm thick coronal sections using a vibratome Leica VT1200S at 4 °C and stored in PBS, or sectioned into 10 μm thick sagittal sections with a cryostat at -20 °C.

For coronal sections immunostaining, the floating sections were blocked in 1% bovine serum albumin, 4% normal donkey serum and 0.3% Triton X-100 for 2 h at room temperature (RT). The sections were incubated overnight in primary antibody solution at 4 °C. Next day, after washing the sections with PBS (5 times, 5 min each), the sections were stained with secondary antibody solution for 2 h at RT. The sections were washed with PBS (5 times, 5 min each) and mounted using Antifade mounting medium with DAPI (4′,6-diamidino-2-phenylindole, Vector Laboratories). All the antibody solutions were made in blocking buffer. The 1st antibodies used in coronal sections immunostaining were mouse-monoclonal anti-Parvalbumin antibody (sigma, P3088, 1:3000), rabbit monoclonal anti-Somatostatin (Abcam, ab111912, 1:400), mouse monoclonal anti-GFAP (Invitrogen, 1:400, MA5-33059), and mouse monoclonal anti-IBA1 (Invitrogen, MA5027726, 1:600). The 2nd antibodies used in coronal sections immunostaining were Donkey anti-Rabbit immunoglobin G (IgG) conjugated with Alexa Fluor 594, Donkey anti-Mouse IgG conjugated with Alexa Fluor 594, Donkey anti-Rabbit IgG conjugated with Cyanine 5, Donkey anti-Mouse IgG conjugated with Cyanine5, Donkey anti-Rat IgG conjugated with Cyanine 5, Donkey anti-Rabbit IgG conjugated with Alexa 488. All the images were taken using slide scanner (ZEISS Axio Scan Z.1) and confocal microscope (Zeiss LSM980) at 20× objective. The images were analyzed using ImageJ and QuPath.

For sagittal sections immunostaining, half brain was fixed in 4% paraformaldehyde overnight, then saturated in 30% sucrose before immunostaining. The brain sample was frozen in Tissue-plus OCT (optimal cutting temperature) Compound (Fisher Scientific 23-730-571) and stored at -80 °C. 10-micron sagittal sections were used for immunohistochemistry study. The following 1st antibodies were used for sagittal section immunostaining: GFAP (Dako Z0334), IBA1 (Cell Signaling 17198 S), Somatostatin (SST) (Fisher Scientific PA5-85759), Alexa Fluor 488 anti-β-Amyloid, 1–16 Antibody (Biolegend, Cat#:803013), Rabbit beta Amyloid Polyclonal Antibody (CT695) (Invitrogen, Catalog # 51-2700), pTau (Ser202/Thr205) antibody (Cell Signaling 30505). The following 2nd antibodies were used for sagittal section immunostaining Goat anti-Rabbit IgG (H + L) Cross-Adsorbed Secondary Antibody, Alexa Fluor 488 (Life Tech Corp A11008), Goat anti-Mouse IgG2a Cross-Adsorbed, Alexa Fluor 647 Life Tech Corp A21241), Goat anti-Rabbit IgG (H + L) Cross-Adsorbed Secondary Antibody, Cyanine3 (Life Tech Corp A10520). After Hoechst staining, sections were imaged using a Keyence microscope and analyzed using Image J.

To reveal misfolded protein aggregates by Thioflavin S, sagittal brain sections were stained with 0.004% Thioflavin S (Sigma-Aldrich T1892-25G) in the dark for 2 h in room temperature or 4°ꓚ overnight in 50% ethanol followed by two washes with 50% ethanol and three washes with PBS. Afterward, sections were mounted for imaging or processed for further immunofluorescent co-staining with other antibodies.

### Statistical analysis

Data are presented as mean ± standard error of the mean (SEM). The significance level was set at *p* < 0.05. The Shapiro–Wilk test was used to assess the normality of the data distribution. For comparisons across behavior tests, such as discrimination indices measured before and after the intervention during NOR and social behavior tests, a two-way repeated-measures Analysis of Variance (ANOVA) was used with group (CT-group vs. PA-group) as the between-subject factor and time (pre-intervention vs. post-intervention) as the within-subject factor. When significant effects were detected, Sidak’s post-hoc test was applied for multiple comparisons with 7 selected pairs including: (1) Pre-test 1 CT-group vs. post-test CT-group; (2) Pre-test 2 CT-group vs. post-test CT-group; (3) Pre-test 3 CT-group vs. post-test CT-group; (4) Pre-test 1 PA-group vs. post-test PA-group; (5) Pre-test 2 PA-group vs. post-test PA-group; (6) Pre-test 3 PA-group vs. post-test PA-group; (7) post-test CT-group vs. post-test PA-group. For within-phase comparisons of individual NOR and social behavior tests, Wilcoxon matched-pairs signed rank test was used. For immunostaining analyses performed after the intervention, comparisons between the CT-group and PA-group were analyzed using an unpaired t-test with Welch’s correction when data followed a normal distribution but with unequal variance. If the data did not meet the normality assumption, the Mann–Whitney U test was used instead.

## Supplementary Information

Supplementary Material 1.

## Data Availability

Requests for further information and resources should be directed to and will be fulfilled by the lead contact, Yun Li (yli30@uwyo.edu).

## Abbreviations

Aβ: Amyloid-beta
AD: Alzheimer’s disease
ANOVA: Analysis of Variance
BDNF: Brain-derived neurotrophic factor
BPSD: Behavioral and psychological symptoms of dementia
CT: Control
DAPI: 4′,6-diamidino-2-phenylindole
DI: Discrimination index
FDA: Food and Drug Administration
GFAP: Glial Fibrillary Acidic Protein
H: Habituation
IACUC: Institutional Animal Care and Use Committee
IBA1: Ionized Calcium-Binding Adapter Molecule 1
IgG: Immunoglobin G
NIH: National Institutes of Health
NOR: Novel object recognition
OCT: Optimal cutting temperature
PA: Physical activity
PBS: Phosphate buffered saline
PFA: Paraformaldehyde
pTau: Hyperphosphorylated tau
PV: Parvalbumin
RT: Room temperature
SB: Sociability
SEM: Standard error of the mean
SN: Social novelty
SST: Somatostatin
Thio S: Thioflavin S
VO_2_ max: The maximal oxygen consumption
WT: Wild type
