# Supplementary material for: Forced treadmill running and ladder climbing physical activity attenuates cognitive decline and protein aggregates in aged 3xTg-AD mice
Source: Mol Brain. 2026 Jul 23;19:69. doi: 10.1186/s13041-026-01332-z (PMC13643970; doi:10.1186/s13041-026-01332-z)
Supplement: Supplementary file 1 [file 13041_2026_1332_MOESM1_ESM.pdf]

Supplemental figures and figure legends

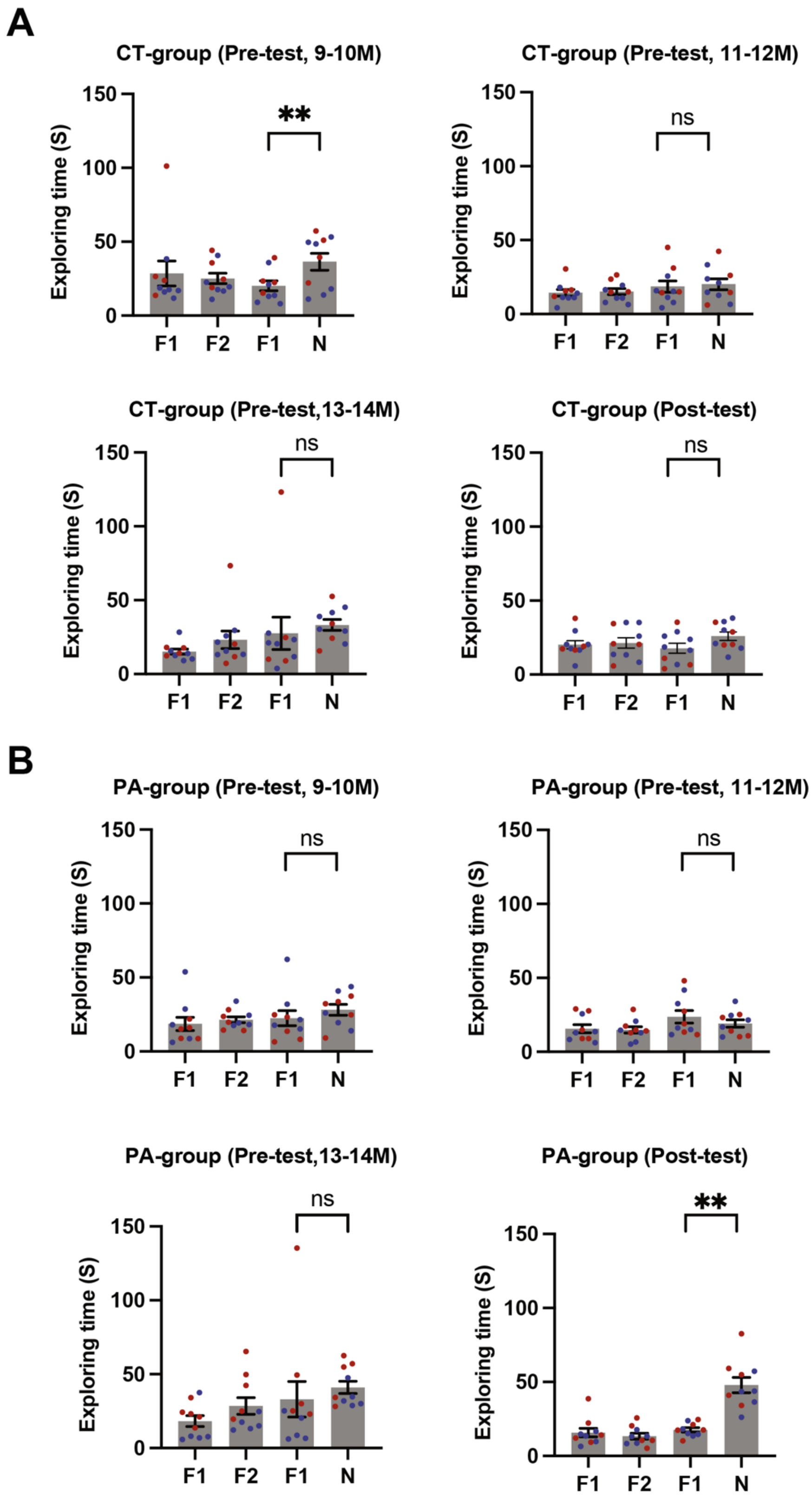

Figure S1. Exploration time for familiar and novel objects in the novel object recognition (NOR) task.

(**A**) Bar graphs display object exploration time in the sedentary control (CT) group, during pre-tests at 9-10, 11-12, and 13-14 months of age and during post-test. For each NOR test, exploration times for two identical familiar objects were measured at the familiarization stage (F1 and F2), and exploration times for the familiar object and novel object were quantified at the testing stage (F and N). At 9-10 months of age, control group displayed significant novel-object preference, indicating a normal recognition memory. After 11-12 months of age, CT-group displayed no preference to the novel object, indicating age-associated decline in recognition memory.

(**B**) Bar graphs display object exploration time in forced treadmill running and ladder climbing physical activity (PA) group, during pre-tests at 9-10, 11-12, and 13-14 months of age and during post-test. Before physical activity intervention, aged 3xTg-AD mice showed impaired preference for the novel object across assessment time points, suggesting compromised recognition memory. Following the 6-week forced treadmill running and ladder climbing physical activity intervention, the PA-group showed significantly greater exploration time of the novel object than the familiar object, whereas the sedentary control group did not show significant novel-object preference at post-test.

Data are presented as mean ± SEM with individual mice data points shown (Blue, male; red, female). Statistical significance was determined by within-time-point Wilcoxon matched-pairs signed rank test comparisons between familiar-object and novel-object exploration during the testing stage. CT-group, sedentary control group; PA-group, forced treadmill running and ladder

climbing physical activity group; F1 and F2, identical familiar objects during familiarization; F, familiar object during testing; N, novel object; ns, not significant; $^{**}p < 0.01$.

A

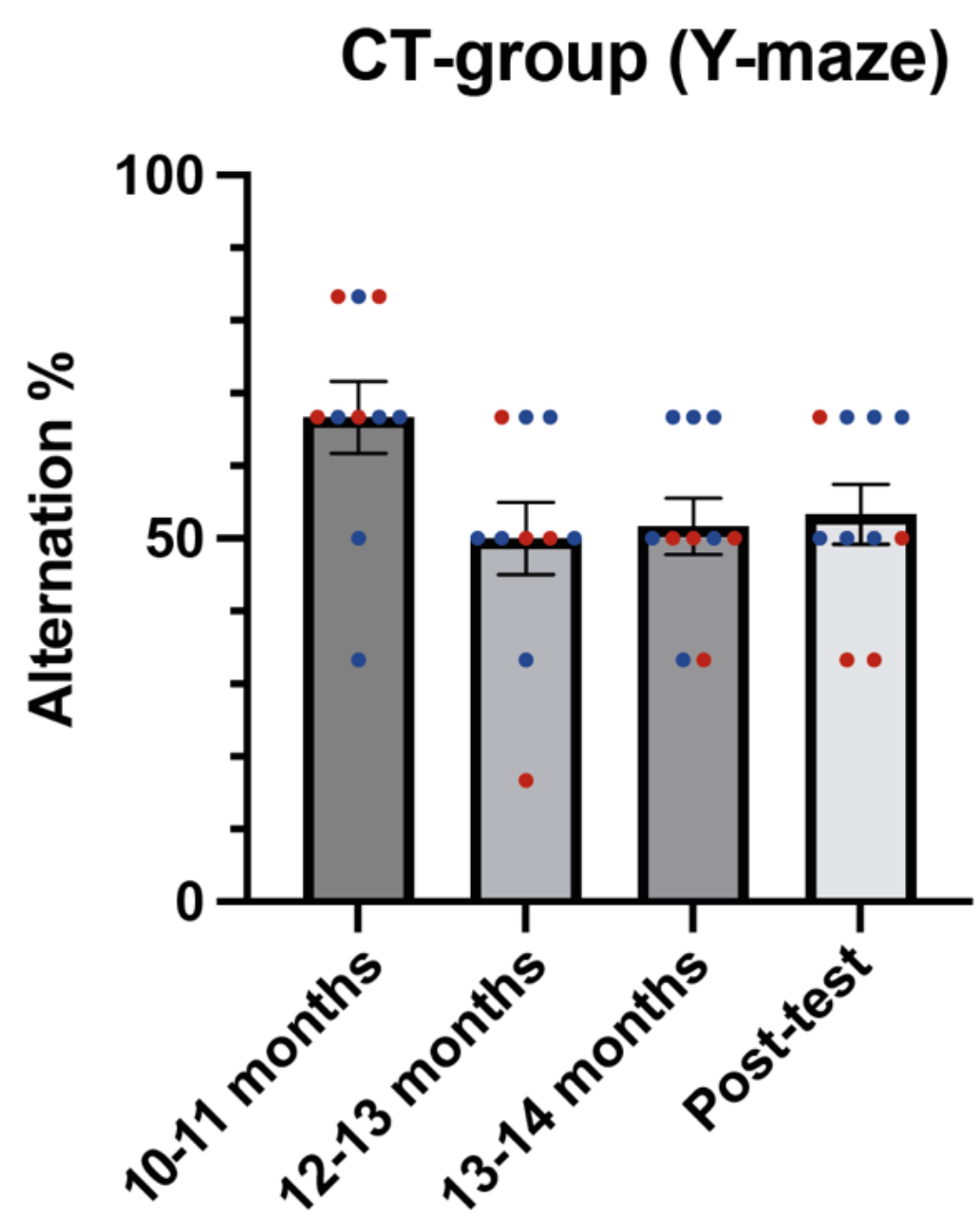
CT-group (Y-maze)
Alternation %
100
50
0
10-11 months
12-13 months
13-14 months
Post-test

B

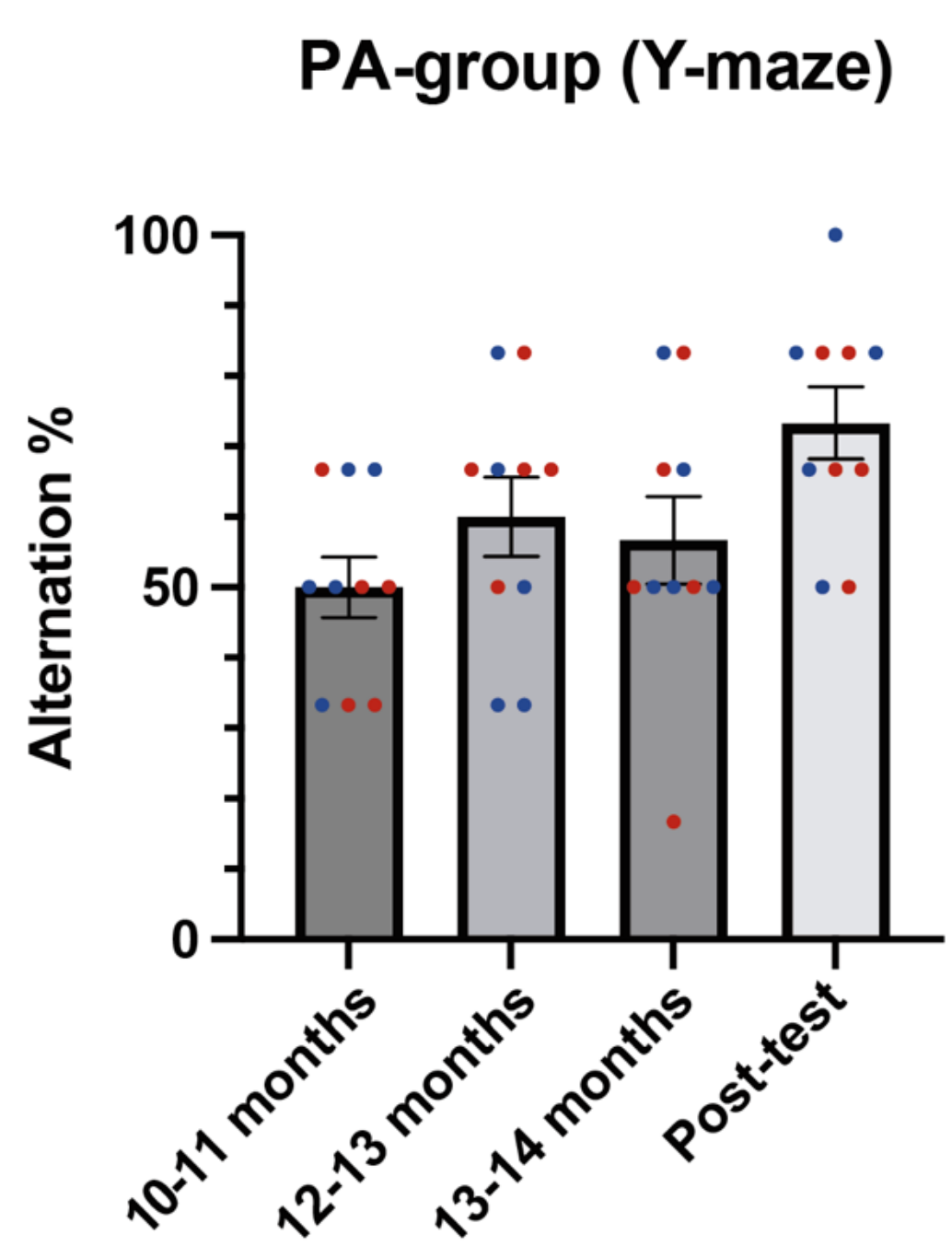
PA-group (Y-maze)
Alternation %
100
50
0
10-11 months
12-13 months
13-14 months
Post-test

Figure S2. Within-group analysis of Y-maze spontaneous alternation performance.

(A) Y-maze spontaneous alternation percentage in the sedentary control (CT) group during pre-tests at 10-11, 12-13, and 13-14 months of age and during post-test. No significant change in alternation performance was detected across time points by one-way ANOVA.

(B) Y-maze spontaneous alternation percentage in the forced treadmill running and ladder climbing physical activity (PA) group during pre-tests at 10-11, 12-13, and 13-14 months of age and during post-test. No significant change in alternation performance was detected across time points by one-way ANOVA.

Data are presented as mean ± SEM with individual mice data points shown (Blue, male; red, female). CT-group, sedentary control group; PA-group, forced treadmill running and ladder climbing physical activity group.

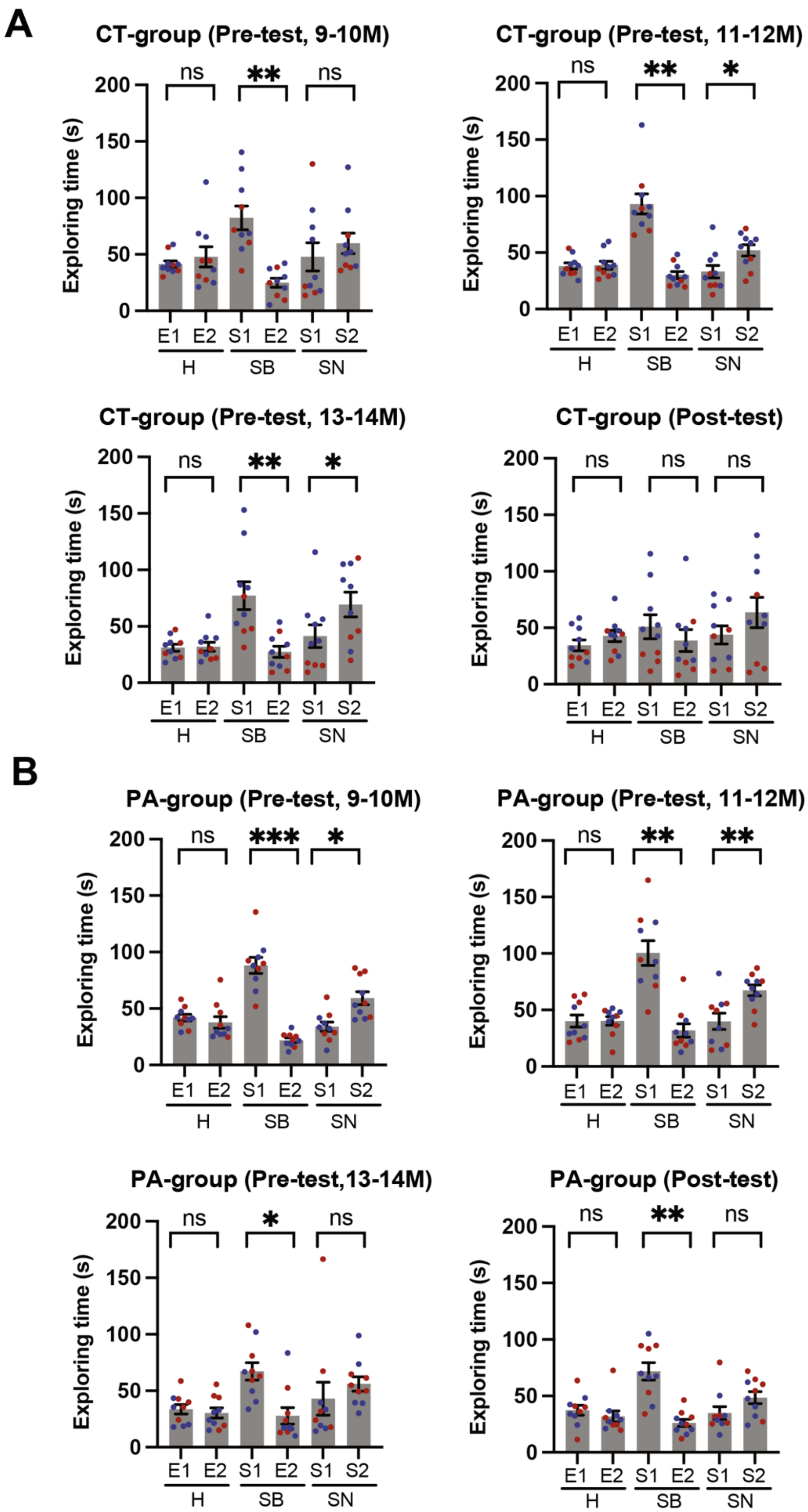
A
CT-group (Pre-test, 9-10M)
CT-group (Pre-test, 11-12M)
CT-group (Pre-test, 13-14M)
CT-group (Post-test)
B
PA-group (Pre-test, 9-10M)
PA-group (Pre-test, 11-12M)
PA-group (Pre-test,13-14M)
PA-group (Post-test)
Exploring time (s)
0
50
100
150
200
ns
*
**
***
E1
E2
S1
E2
S1
S2
H
SB
SN

Figure S3. Exploration times for social behavior test.

(A) Exploration times in the sedentary control (CT) group during pre-tests at 9-10, 11-12, and 13-14 months of age and during post-test. Each test contains the habituation, sociability, and social novelty stages. 3xTg-AD mice showed normal sociability across all pre-tests, and normal social novelty in most pre-tests. During post-test (16-17 months of age), the sedentary control group showed abnormal sociability and abnormal social novelty, suggesting age-related deterioration in social behavioral performance.

(B) Exploration times in the forced treadmill running and ladder climbing physical activity (PA) group during pre-tests at 9-10, 11-12, and 13-14 months of age and during post-test. Each test contains the habituation, sociability, and social novelty stages. Before intervention, 3xTg-AD mice showed normal sociability across all pre-tests, and normal social novelty in most pre-tests. After the 6-week physical activity intervention, mice displayed preserved sociability but abnormal social novelty, suggesting a protective effect on sociability by physical activity intervention.

Data are presented as mean ± SEM with individual mice data points shown (Blue, male; red, female). Statistical significance was determined by Wilcoxon matched-pairs signed rank test within-phase comparisons for habituation (between E1 and E2), sociability (between S1 and E2) and social novelty (between S1 and S2) stages at each time point. CT-group, sedentary control group; PA-group, forced treadmill running and ladder climbing physical activity group; H, habituation; SB, sociability; SN, social novelty; E1 and E2, empty wire cages; S1, first-introduced social target; S2, second-introduced novel social target; ns, not significant; $^{*}p < 0.05$; $^{**}p < 0.01$; $^{***}p < 0.001$.

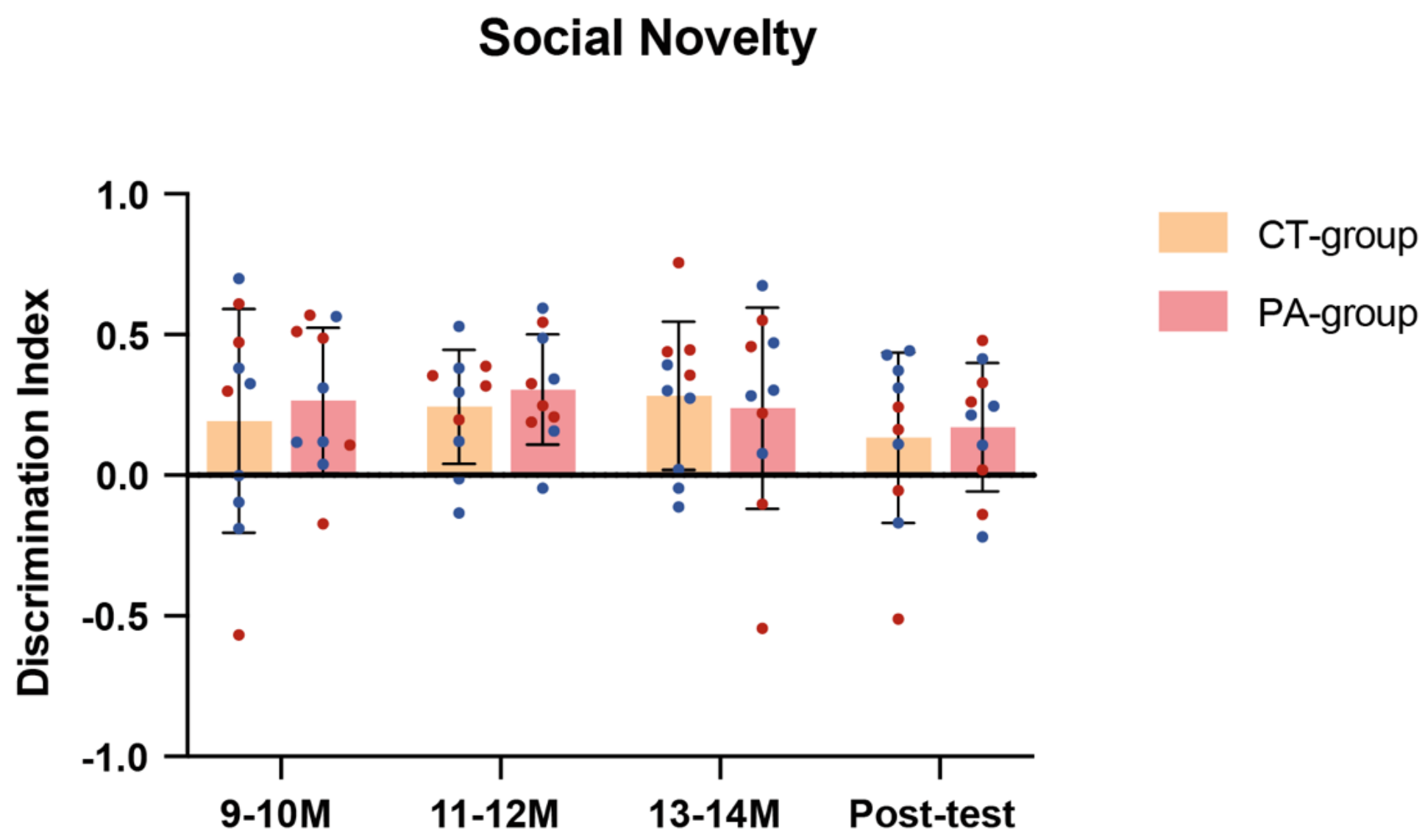

Figure S4. Social novelty discrimination was not significantly altered by physical activity. Discrimination index during the social novelty stage of the social behavior test in sedentary control (CT) and forced treadmill running and ladder climbing physical activity (PA) groups during pre-tests at 9–10, 11–12, and 13–14 months of age and during post-test. The discrimination index was calculated to evaluate preference for the novel social target over the familiar social target. No significant group differences were detected across the assessment period.

Data are presented as mean ± SEM with individual mice data points shown (Blue, male; red, female). Statistical significance was determined using two-way repeated-measures ANOVA followed by Sidak’s multiple comparisons test. CT-group, sedentary control group; PA-group, forced treadmill running and ladder climbing physical activity group.

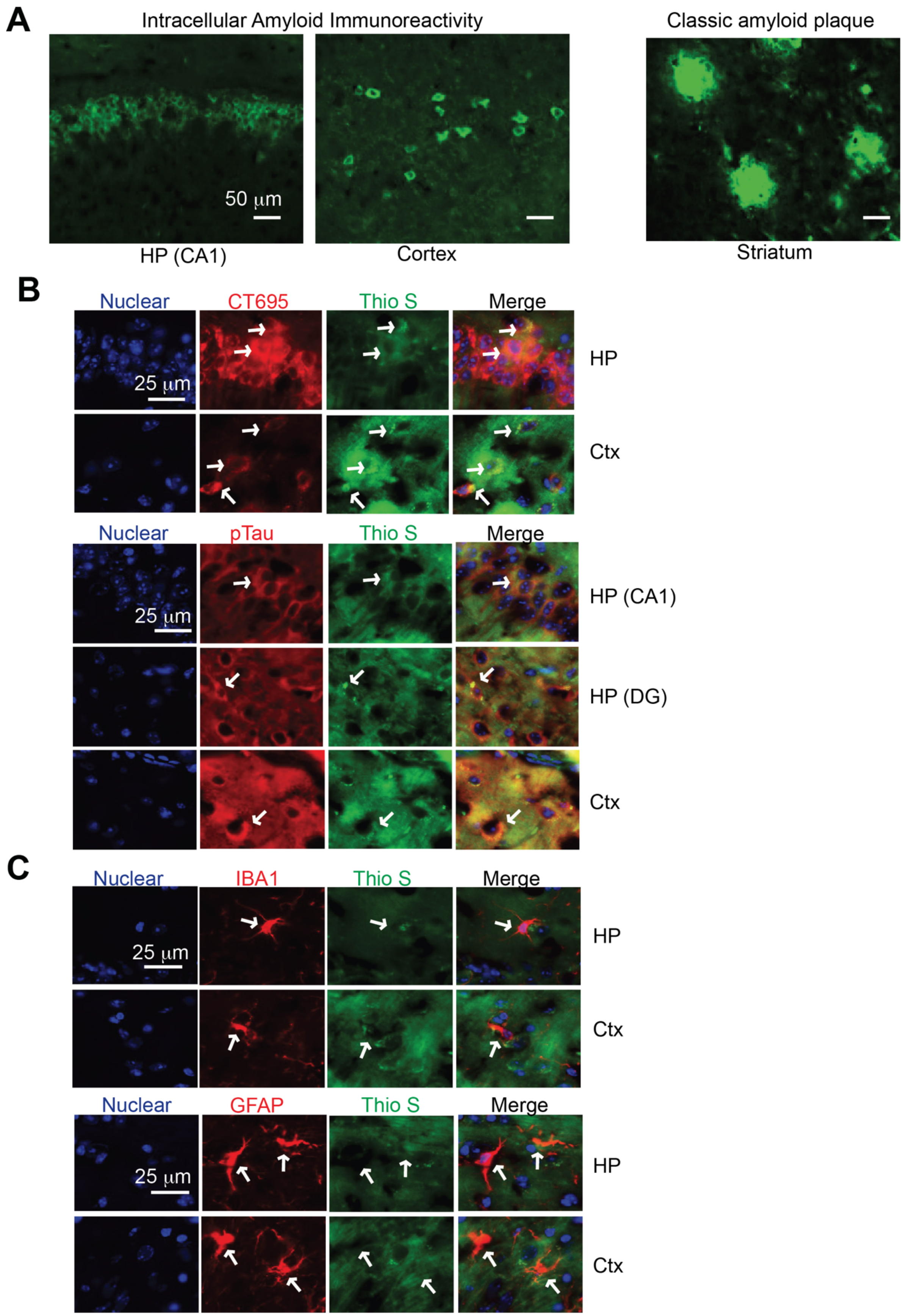

A
Intracellular Amyloid Immunoreactivity
50 µm
HP (CA1)
Cortex
Classic amyloid plaque
Striatum
B
Nuclear
CT695
Thio S
Merge
25 µm
HP
Ctx
Nuclear
pTau
Thio S
Merge
25 µm
HP (CA1)
HP (DG)
Ctx
C
Nuclear
IBA1
Thio S
Merge
25 µm
HP
Ctx
Nuclear
GFAP
Thio S
Merge
25 µm
HP
Ctx

Figure S5. Amyloid staining patterns in brain regions of aged 3xTg-AD mice.

(A) Representative images of β-Amyloid immunohistochemistry using Alexa Fluor 488 anti-β-Amyloid, 1-16 Antibody (Biolegend, Cat#:803013). Left and middle panels: intracellular amyloid immunoreactivity in the hippocampus (CA1) and cortex area. Right panel: classic amyloid plaques immunohistochemistry in the striatum.

(B) Representative images showing Thio S colocalized with both Amyloid and pTau. Top panels: Images of β-Amyloid antibody CT695 (Invitrogen, Catalog # 51-2700) co-staining with Thio S immunohistochemistry in the hippocampus (HP) and cortex (Ctx)area. Bottom panels: Images of pTau co-staining with Thio S immunohistochemistry in the hippocampus (HP) and cortex (Ctx) area. White arrows indicate Thio S, amyloid specific antibody, and pTau positive staining signal.

(C) Representative images showing Thio S doesn't colocalize with glial markers. Top panels: Images of IBA1 co-staining with Thio S immunohistochemistry in the hippocampus (HP) and cortex (Ctx) area. Bottom panels: Images of GFAP co-staining with Thio S immunohistochemistry in the hippocampus (HP) and cortex (Ctx) area. White arrows indicate Thio S, IBA1, or GFAP positive staining signals.

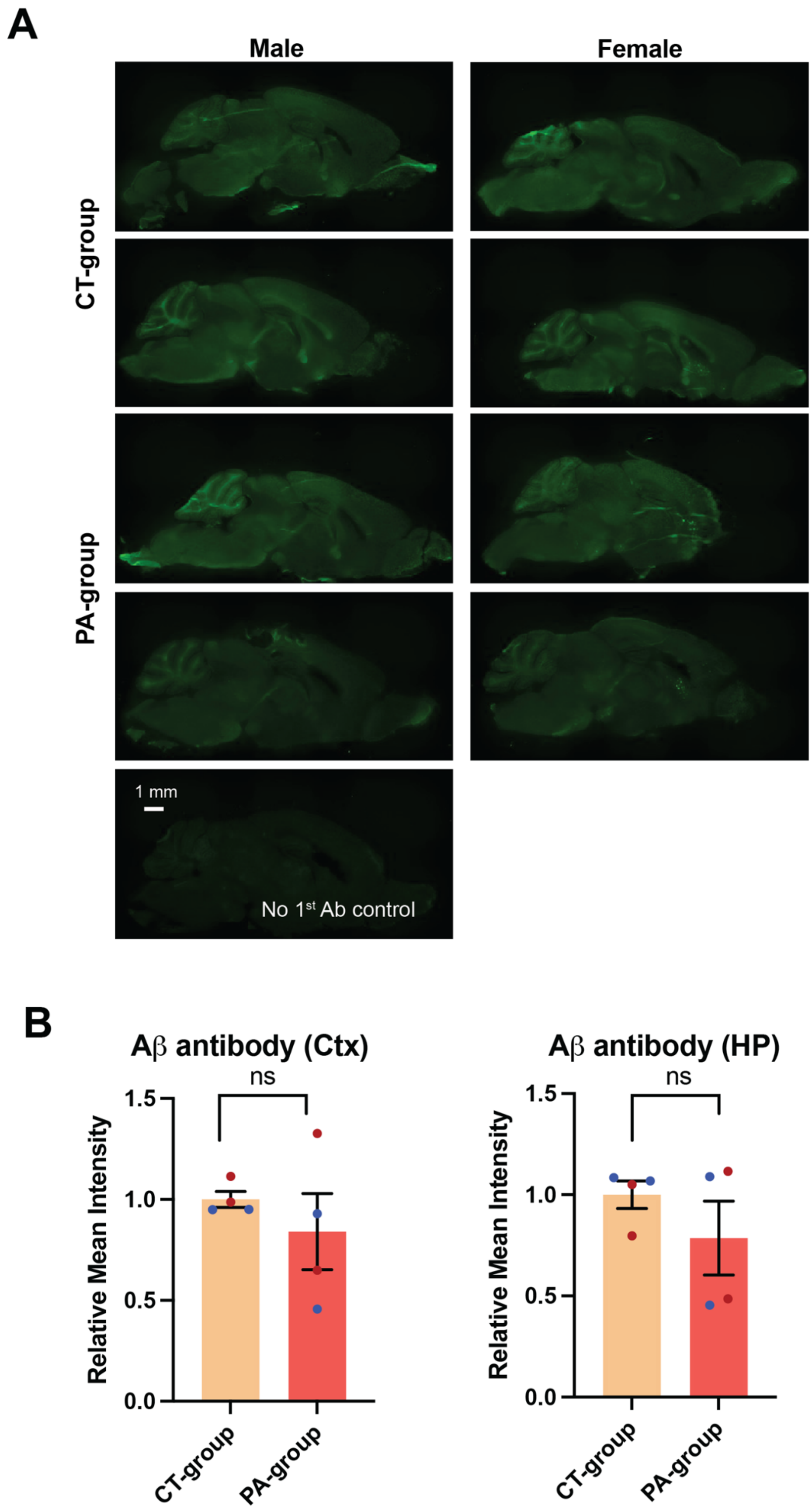
A
Male
Female
CT-group
PA-group
1 mm
No 1st Ab control
B
Aβ antibody (Ctx)
Aβ antibody (HP)
ns
ns
Relative Mean Intensity
1.5
1.0
0.5
0.0
CT-group
PA-group

Figure S6. Forced treadmill running and ladder climbing physical activity did not change amyloid immunostaining intensity detected by amyloid specific antibody in the cortex and hippocampus of aged 3xTg-AD mice.

(A) Representative Images of β-Amyloid immunohistochemistry using Alexa Fluor 488 anti-β-Amyloid, 1-16 Antibody (Biolegend, Cat#:803013) in sagittal brain sections of this study. Representative images from both sexes are shown for each experimental group. A no-primary-antibody control section is included to demonstrate background fluorescence. Scale bar = 1 mm.

(B) Quantification of relative mean intensity of β-Amyloid immunohistochemistry comparison between CT-group and PA-group in the cortex and hippocampus.

Data are presented as mean ± SEM with dots representing individual mice (Blue, male; red, female). Statistical significance was determined using two-tailed unpaired t-test with Welch's correction. CT-group, sedentary control group; PA-group, forced treadmill running and ladder climbing physical activity group; Ctx, cortex; HP, hippocampus; ns, not significant.

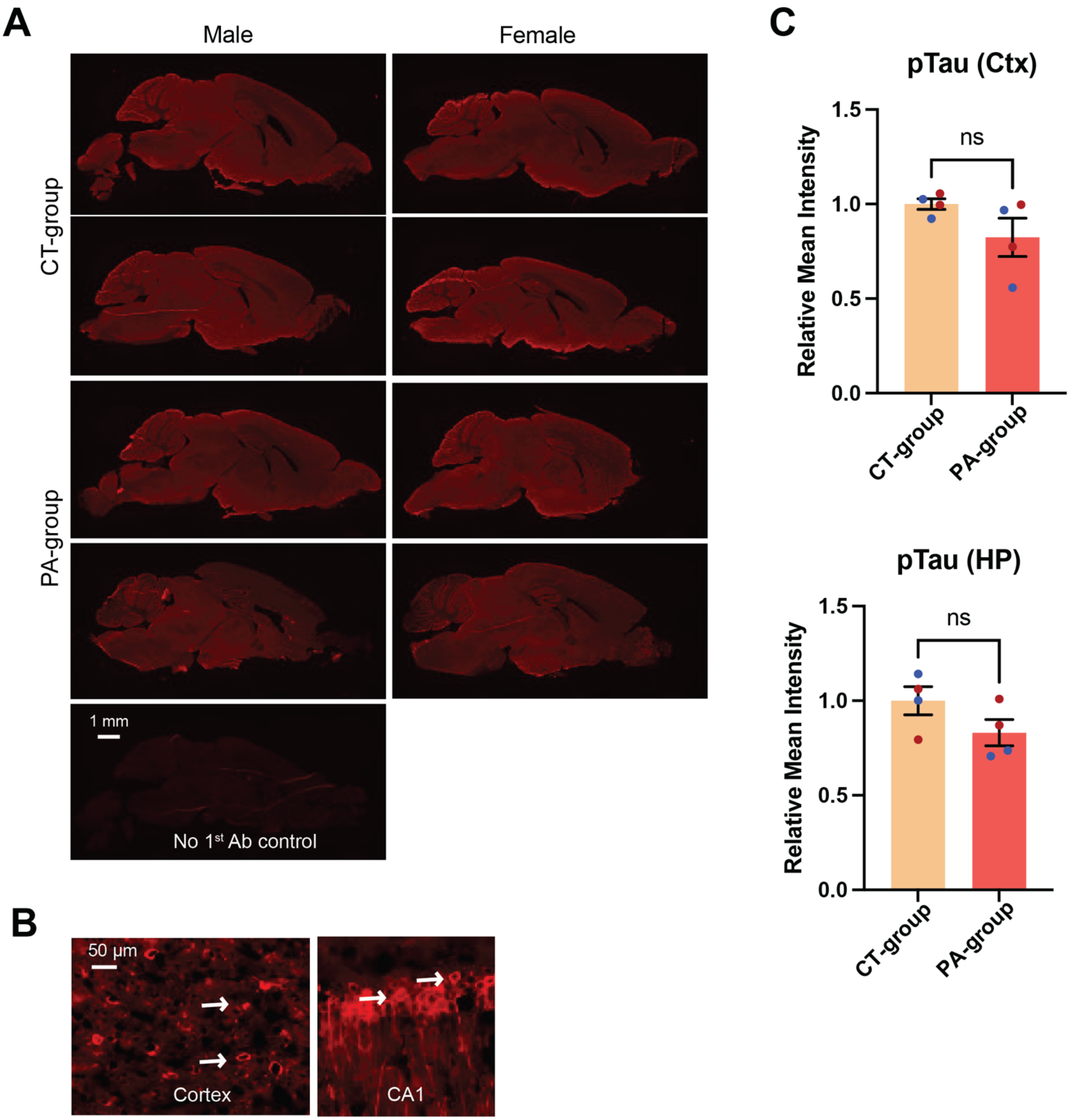
A
Male
Female
CT-group
PA-group
1 mm
No 1st Ab control
B
50 µm
Cortex
CA1
C
pTau (Ctx)
Relative Mean Intensity
ns
1.5
1.0
0.5
0.0
CT-group
PA-group
pTau (HP)
Relative Mean Intensity
ns
1.5
1.0
0.5
0.0
CT-group
PA-group

Figure S7. Forced treadmill running and ladder climbing physical activity did not change pTau intensity in the cortex and hippocampus of aged 3xTg-AD mice.

(A) Representative sagittal brain section images of pTau immunohistochemistry, including the sedentary CT-group (n = 4: male n = 2, female n = 2), PA-group (n = 4: male n = 2, female n = 2) respectively. No 1st antibody control was shown as well. Scale bar = 1 mm.

(B) Representative pTau images with higher magnification in the cortex and hippocampus CA1 region. White arrows indicate pTau positive staining signal. Scale bar = 50 μm.

(C) Quantification of mean intensity of pTau comparison between CT-group and PA-group in the cortex and hippocampus.

Data are presented as mean ± SEM with individual mice data points shown (Blue, male; red, female). Statistical significance was determined using two-tailed unpaired t-test with Welch's correction. CT-group, sedentary control group; PA-group, forced treadmill running and ladder climbing physical activity group. ns, not significant.

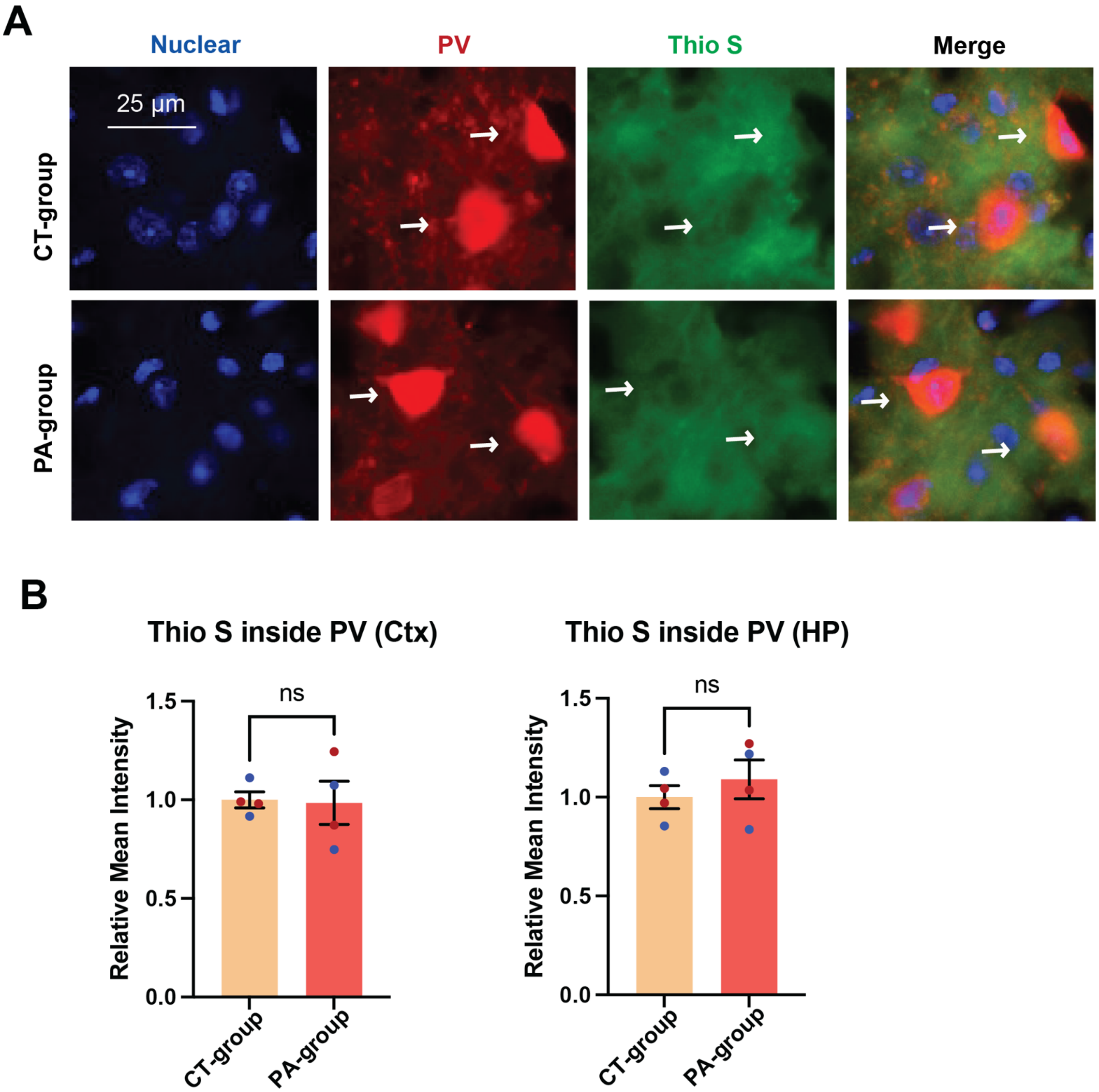

A
Nuclear
PV
Thio S
Merge
25 µm
CT-group
PA-group
B
Thio S inside PV (Ctx)
Thio S inside PV (HP)
ns
ns
Relative Mean Intensity
1.5
1.0
0.5
0.0
CT-group
PA-group

Figure S8. Forced treadmill running and ladder climbing physical activity did not change Thioflavin S accumulation in PV-positive interneurons of aged 3xTg-AD mice.

(A) Representative fluorescence images of brain sections stained for nuclei, PV, and Thioflavin S in sedentary control and forced treadmill running and ladder climbing physical activity groups. Merged images illustrate the distribution of Thioflavin S signal relative to PV-positive regions. White arrows indicate regions with overlapping PV-positive inhibitory interneurons and Thioflavin S–positive signal. Scale bar = 25 μm.

(B) Quantitative analysis of relative Thioflavin S mean fluorescence intensity within PV-positive interneurons in the cortex and hippocampus. Forced treadmill running and ladder climbing physical activity did not change Thioflavin S intensity within PV-positive interneurons in both the cortex and hippocampus compared with sedentary controls.

Data are presented as mean ± SEM with dots representing individual mice (Blue, male; red, female). Statistical significance was determined using two-tailed unpaired t-test with Welch's correction. CT-group, sedentary control group; PA-group, forced treadmill running and ladder climbing physical activity group; Ctx, cortex; HP, hippocampus; PV, parvalbumin; Thio S, Thioflavin S; ns, not significant.
